# Methodological and reporting quality of systematic and rapid reviews on human mpox and their utility during a public health emergency

**DOI:** 10.1002/cesm.70005

**Published:** 2024-11-15

**Authors:** Kusala Pussegoda, Izza Israr, Austyn Baumeister, Tricia Corrin, Melanie Sterian, Mavra Qamar, Anmol Samra, Lisa Waddell

**Affiliations:** ^1^ Public Health Risk Sciences Division, National Microbiology Laboratory, Public Health Agency of Canada Guelph Ontario Canada; ^2^ Department of Population Medicine University of Guelph Guelph Ontario Canada

**Keywords:** evidence mapping, methodological quality, mpox, rapid review, reporting quality, scoping review, systematic review

## Abstract

**Introduction:**

Evidence syntheses were rapidly produced during the 2022 mpox outbreak despite a lack of studies. The aim of this methodological study was to assess the quality and utility of the evidence syntheses produced during the first 6 months of the outbreak compared to those published before it.

**Methods:**

Human mpox evidence syntheses available before December 31, 2022 were retrieved from PubMed, Scopus, EuropePMC, SSRN, and arXiv. Study characteristics, utility, methodological, and reporting quality (AMSTAR‐2 and PRISMA) were contrasted between syntheses produced before the 2022 outbreak (historical) and during the first 6 months (new). Results were synthesized narratively.

**Results:**

Twenty‐six evidence syntheses were included; two historical systematic reviews (SRs) and 24 new SRs, rapid reviews, scoping reviews, and mislabelled syntheses. Median time from search to publication/preprint post date was 68 and 6 weeks for historical and new syntheses, respectively. Among the new syntheses, 8% (2/24) did not include evidence from the 2022 outbreak, 33% (8/24) included only new evidence and 58% (14/24) included both new and historical evidence. Only 29% of new syntheses contrasted findings between new and historical evidence. Methodological quality was critically low for 100% of historical syntheses and 92% of new syntheses and the remainder (8%) were low. Reporting quality was poor with a median of 10.5 (range 10–11) and 11.5 (range 4–21) of 27 items reported sufficiently by historical and new syntheses, respectively.

**Conclusions:**

Evidence syntheses take time to produce and during an emergent outbreak they are often outdated at the time of publication and suffer from poor adherence to methodological and reporting guidelines. Overlapping content and few new studies resulted in minimal added value to the mpox literature. Strategies to reduce duplication and mechanisms to produce and disseminate continuously updated living evidence syntheses need to be explored to support decision‐makers responding to an emergency.

## 1 INTRODUCTION

During an emergent public health outbreak such as COVID‐19 and mpox, there is a rapid increase in literature and evidence is constantly evolving, making it difficult for public health decision‐makers to stay up‐to‐date with the most current evidence [1, 2, 3]. Decision‐makers need timely synthesis of evidence on a wide range of topic areas to understand what is known and unknown to inform on‐going response activities, particularly during the early phase of an outbreak.

Mpox was discovered in the Democratic Republic of Congo in 1970. Before the 2022 outbreak, mpox was largely contained to central and western Africa and cases were occasionally exported to non‐endemic countries. Two systematic reviews (SRs) were published before 2022 that summarized the epidemiology of human mpox from the primary literature with evidence up to 2020 [4, 5]. Many conventional evidence syntheses including SRs, rapid reviews (RRs), and scoping reviews (ScRs) were initiated at the beginning of the 2022 mpox outbreak to rapidly synthesize the evolving evidence from the outbreak despite a lack of new high‐quality primary studies [2].

Utility of evidence syntheses depends on methodological rigor (i.e., how well the study is designed and conducted) [6], transparency in reporting (i.e., how well methods and finding are described) [7], timeliness, and whether the review addresses relevant questions. These are critical for end‐users, to be confident in the findings and interpretation of the results to inform response activities during a public health emergency [8, 9]. However, review authors should consider the value of conducting conventional evidence syntheses during an emergent outbreak when evidence is emerging rapidly. Decision‐makers need to know “what is known” at the time of inquiry during an outbreak, and even the fastest syntheses are likely to be out‐of‐date by the time they are published [10, 11]. Framing the issues around the conduct of conventional evidence syntheses during a public health emergency can drive discussion around solutions for the synthesis of evidence during an emergent outbreak, when continuously synthesized evidence is urgently needed by decision‐makers.

This study aims to evaluate the utility of evidence syntheses during the first 6 months of the 2022 mpox outbreak and to examine their adherence to reporting and methodological quality guidelines. The findings will add evidence to define the issue and support development of a framework for evidence syntheses practices during public health emergencies.

## 2 METHODS

A protocol was developed *a priori* by the authors who have expertise in knowledge syntheses, epidemiology, infectious diseases, and public health (File S1). Minor deviations were made to evaluate methodological quality and are outlined below.

The study research questions were: (1) What is the methodological and reporting quality of the evidence syntheses on human mpox studies conducted during the first 6 months of the 2022 outbreak and how does it compare to evidence syntheses conducted before the outbreak? (2) What was the purpose and utility of conducting evidence syntheses on human mpox studies during the first 6 months of the outbreak?

### 2.1 Eligibility criteria

Evidence syntheses on mpox in human populations from inception to December 31, 2022 were included if they met the minimum criteria outlined in the Preferred Reporting Items for Systematic Reviews and Meta Analyses for protocols (PRISMA‐P) definition of a SR [7, 12]. That is, reviews explicitly stating methods to identify studies (i.e., search strategy methods and search dates), methods used for study selection (i.e., eligibility criteria and study selection process), and a narrative or quantitative synthesis of evidence. Included evidence syntheses were restricted to those published in English and French due to human resource limitations. Exclusions included primary literature and evidence syntheses on nonhuman mpox populations, narrative reviews, overviews of reviews, and other nonprimary literature (File S1).

### 2.2 Search

As part of a separate literature surveillance project for mpox, a search strategy was developed to establish a database of all mpox literature as described previously [2]. Briefly, PubMed, Scopus, EuropePMC, SSRN, and arXiv were searched twice weekly between May 1, 2022 and December 31, 2022 utilizing keywords (e.g., monkeypox, mpox, simianpox) with no language restrictions (File S1). All citations previously screened as non‐primary during the mpox literature surveillance project were re‐screened to identify evidence syntheses on human outcomes. Evidence syntheses on the 2022 mpox outbreak published up to December 31, 2022 were included. A secondary search from database inception to April 30, 2022 was conducted to identify syntheses published before the 2022 outbreak and included additional keywords to target evidence syntheses (e.g., SRs, ScRs, and meta‐analyses [MA])). Grey literature and reference lists of relevant evidence syntheses were searched to identify omitted relevant syntheses (File S1).

### 2.3 Data management

Search results were collated in Endnote v20 (Clarivate) and transferred to DistillerSR (Evidence Partners 2023) to manage relevance screening and data extraction. Duplicates were identified and removed in each software.

### 2.4 Study selection

Eligibility screening forms were developed *a priori* and pilot tested before screening (File S1). Titles/abstracts and full text screening were conducted independently by two reviewers (II, KP, TC, AS, MQ, AB, and MK) and disagreements were resolved by consensus or by consulting a senior reviewer (LW or KP).

### 2.5 Data extraction

A data extraction form was developed *a priori* and pilot tested by reviewers (File S1). Extraction was conducted independently by two reviewers and resolved by consensus or adjudication with a senior reviewer. Study characteristics (e.g., language, article type, and number of databases searched) were extracted. The following characteristics were used to evaluate utility of evidence syntheses: number of included studies, what type of studies were included (e.g., pre‐2022 outbreak or post‐2022 outbreak), whether syntheses compared 2022 outbreak and pre‐2022 outbreak evidence, timeliness of publication, preprints of published syntheses availability, topics/outcomes covered and overlap across new and historical evidence syntheses, disclosure of a previously published synthesis covering the topic/outcome, reporting of study limitations, and type of evidence (e.g., descriptive vs. analytical observational studies) included in the evidence syntheses.

### 2.6 Methodological and reporting quality

Assessment of methodological and reporting quality was conducted independently by two reviewers and disagreements were resolved by consensus or third‐party adjudication with a senior reviewer (File S1).

Methodological quality was assessed using A Measurement Tool to Assess Systematic Reviews 2.0 (AMSTAR‐2) and adapted for RR and ScRs as no established tools exist to evaluate their methodological quality [6]. AMSTAR‐2 consists of 16 domains including seven critical domains (i.e., domains 2, 4, 7, 9, 11, 13, and 15). A post‐hoc decision was made before analysis to consider publication bias (domain 15) as a noncritical domain since many included studies did not perform a MA and of those that did, there was a lack of power due to the limited number of included studies to investigate publication bias. Each item was evaluated as “yes,” “partial yes,” “no,” or “not applicable” (NA) for items related to MA. Domains 9 and 13 were “NA” for syntheses that did not conduct risk of bias (ROB), similar to previously published studies [13, 14]. Overall quality was classified as “critically low,” “low,” “moderate,” or “high”.

Reporting quality of SRs, MAs, and RRs were evaluated using the PRISMA 2020 statement [15], ScRs using PRISMA‐ScR extension [16], and abstracts using PRISMA‐Abstracts extension [15]. A posthoc decision was made to reclassify studies that were incorrectly named by review authors into appropriate study design categories assessed by our team so the correct evaluation tools were applied for the analysis of reporting quality. All items were evaluated using the response options “yes,” “no,” and “NA.” Each item considered to be sufficiently reported based on the guideline was a “yes” and designated one count. For each item with subitems, an overall “yes” was designated if all subitems were reported.

### 2.7 Analysis

Study characteristics, utility, methodological quality, and reporting quality were synthesized narratively and summarized using descriptive statistics. Characteristics, utility, and methodological quality were analysed as a whole and by type of evidence synthesis as reported by review authors (i.e., SRs, MAs, ScRs, RRs, and other evidence synthesis) while reporting quality was analyzed by type of evidence as assessed by our team. Syntheses published before May 1, 2022 (historical) were contrasted with syntheses published May 1, 2022 onwards (new). Syntheses that were published by December 2022 and were posted earlier as preprints were evaluated for utility and quality using the preprint version which was the version that would have been first available for use by decision‐makers. Preprint and published versions of synthesis published by December 2022 were contrasted post hoc to evaluate if there were changes to the utility and quality. The complete data set is available on Open Science Framework (https://osf.io/4MEA6/).

## 3 RESULTS

**Figure 1:**
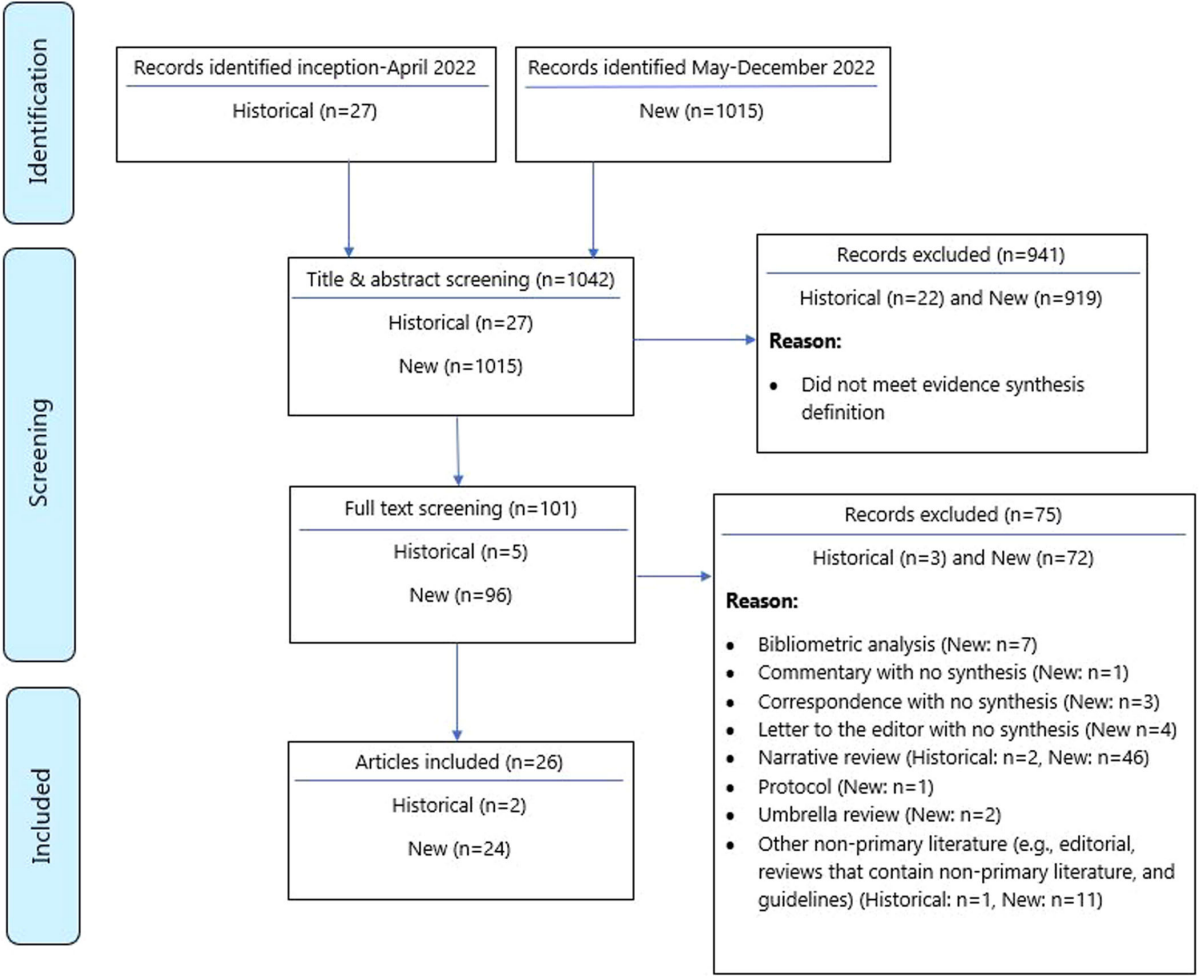
PRISMA flow diagram of the mpox syntheses during the first 6 months of the outbreak.

A total of 1015 articles were identified from the twice weekly literature surveillance between May 1, 2022 and December 31, 2022 and an additional 27 evidence syntheses were identified from inception to April 30, 2022. Twenty‐six evidence syntheses were included, of which two were historical and 24 were new (Figure 1). Historical syntheses were classified as SRs (*n* = 2) and new syntheses as SR/SR‐MA (*n* = 14), ScR (*n* = 4), RR (*n* = 2), MA (*n* = 1), and other unconventional labels (*n* = 5) (Table 1).

**Table 1:** Characteristics of included syntheses.

Characteristic	Categorization	Systematic review ± meta‐analysis		Meta‐analysis (*N* = 1)	Scoping review (*N* = 4)	Rapid review (*N* = 2)	Other^a^ (*N* = 5)	Total new syntheses (*N* = 24)
		Historical (*N* = 2)	2022 outbreak (*N* = 12)					
Country of first author (*n*, %)	Algeria				1 (25%)			1 (4%)
	Brazil		1 (8%)	1 (100%)			2 (40%)	4 (17%)
	Colombia						1 (20%)	1 (4%)
	Egypt		1 (8%)				1 (20%)	2 (8%)
	India		2 (16%)					2 (8%)
	Iran		1 (8%)					1 (4%)
	Italy		1 (8%)					1 (4%)
	Kenya				1 (25%)			1 (4%)
	Nepal		1 (8%)					1 (4%)
	Netherlands	1 (50%)						0 (0%)
	Peru		3 (25%)		1 (25%)			4 (17%)
	Spain					1 (50%)		1 (4%)
	United Kingdom	1 (50%)	1 (8%)					1 (4%)
	United States		1 (8%)		1 (25%)	1 (50%)	1 (20%)	4 (17%)
Type of report (*n*, %)	Peer‐reviewed journal article	2 (100%)	6 (50%)	1 (100%)	2 (50%)	1 (50%)	2 (40%)	12 (50%)
	Preprint		5 (42%)		1 (25%)		2 (40%)	8 (33%)
	Short Communication				1 (25%)	1 (50%)		2 (8%)
	Letter to the Editor						1 (20%)	1 (4%)
	Commentary/Correspondence		1 (8%)					1 (4%)
Language (*n*, %)	English	2 (100%)	12 (100%)	1 (100%)	4 (15%)	2 (100%)	5 (100%)	24 (100%)
Median No. of authors (range)		4.5 (2–7)	7 (4–17)	3 (3–3)	6.5 (1–11)	4 (2–6)	10 (3–18)	6.5 (1–18)
Median No. of databases searched (range)		3 (2‐4)	4 (1–8)	3 (3–3)	4.5 (1–6)	2.5 (2–3)	5 (1–5)	4 (1–7)
Bibliographic database searched (*n*, %)	PubMed	1 (50%)	9 (75%)	1 (100%)	3 (75%)	2 (100%)	5 (100%)	21 (88%)
	PubMed Central						1 (20%)	1 (4%)
	Scopus		8 (67%)	1 (100%)	2 (50%)	1 (50%)	3 (60%)	15 (63%)
	MEDLINE	1 (50%)	6 (50%)		1 (25%)		1 (20%)	8 (33%)
	Web of Science		6 (23%)	1 (100%)	1 (25%)	1 (50%)	2 (40%)	11 (46%)
	Cochrane Library		3 (25%)		2 (50%)			5 (21%)
	Science Direct		2 (16%)		1 (25%)		1 (20%)	4 (17%)
	Embase	2 (100%)	8 (67%)		2 (50%)	1 (50%)	1 (20%)	12 (50%)
	PsychINFO		1 (8%)					1 (4%)
	ProQuest		1 (8%)					1 (4%)
	Other (AMED, LILACS, SCIELO, Hinari, EBSCOHost‐Academic, ilissAfrica, AJOL, Google Scholar, VHL)	1 (50%)	3 (25%)		1 (25%)		3 (60%)	7 (29%)
%Preprint database searched (*n*, %)	medRxiv		2 (16%)		1 (25%)			3 (13%)
	Research Square		1 (8%)		1 (25%)			2 (8%)
	OSF preprints				1 (25%)			1 (4%)
	Other (manually searched medRxiv, Research Square, and Lancet preprints or did not specify preprint databases)		2 (16%)					2 (8%)
Mislabeled synthesis (*n*, %)	Yes		4 (33%)		1 (25%)	1 (50%)	5 (100%)	11 (46%)
	No	2 (100%)	8 (67%)	1 (100%)	3 (75%)	1 (50%)		13 (54%)
Grey literature search conducted (*n*, %)	Yes	2 (100%)	2 (16%)		1 (25%)		3 (60%)	6 (25%)
	No				1 (25%)			1 (4%)
	Not reported		10 (83%)	1 (100%)	2 (50%)	2 (100%)	2 (40%)	17 (71%)
Grey literature sources searched (*n*, %)	WHO (website and news reports)	1 (50%)	2 (16%)				1 (20%)	3 (13%)
	US CDC (website and news reports)	1 (50%)	1 (8%)				1 (20%)	2 (8%)
	Africa CDC & Nigeria CDC	1 (50%)						0 (0%)
	European Centre for Disease Prevention and Control	1 (50%)					1 (20%)	1 (4%)
	Other (African Field Epidemiology Network, EpiCentre, ProMed, ICMR, leading newsprint articles)	1 (50%)	1 (8%)					1 (4%)
	Not reported						1 (20%)	1 (4%)
Other sources of evidence searched (*n*, %)	Yes	1 (50%)	9 (75%)		3 (75%)	2 (100%)	3 (60%)	17 (58%)
	Not reported	1 (50%)	3 (25%)	1 (100%)	1 (25%)		2 (40%)	7 (29%)
Other sources of evidence searched (*n*, %)	Manual search by medical librarian					1 (50%)		1 (4%)
	Reference chain approach	1 (50%)	8 (67%)		2 (50%)	1 (50%)	3 (60%)	14 (58%)
	“similar articles” on PubMed		1 (8%)		1 (25%)			2 (8%)
	Backwards citation				1 (25%)			1 (4%)
	Search on Google	1 (50%)	1 (8%)					1 (4%)
	Contacted authors in the field		1 (8%)					1 (4%)
	Not reported	1 (50%)	3 (25%)	1 (100%)	1 (25%)		2 (40%)	7 (29%)
Population of focus in the evidence synthesis (*n*, %)	General population	2 (100%)	11 (92%)	1 (100%)	4 (100%)	1 (50%)	5 (100%)	22 (92%)
	Gay, bisexual, men who have sex with men (gbMSM)		2 (16%)					2 (8%)
	Pregnant women		1 (8%)					1 (4%)
	Healthcare workers		1 (8%)		1 (25%)	1 (50%)		2 (8%)
	Other (university students, medical students)				1 (25%)			1 (4%)
Language restrictions in study selection (*n*, %)	Yes	1 (50%)	4 (33%)		3 (75%)	1 (50%)	2 (40%)	10 (42%)
	No	1 (50%)	6 (50%)		1 (25%)	1 (50%)	2 (40%)	10 (42%)
	Not reported		2 (16%)	1 (100%)			1 (20%)	4 (15%)
Meta‐analysis conducted (*n*, %)	Yes	1 (50%)	6 (50%)	1 (100%)		1 (50%)	2 (40%)	10 (50%)^b^
	No/not reported	1 (50%)	6 (50%)			1 (50%)	3 (60%)	10 (50%)^b^
	Not part of methodology				4 (100%)			N/A
Funding Source (*n*, %)	Pharmaceutical company	1 (50%)						0 (0%)
	Government					2 (100%)		2 (8%)
	Academic and hospital institutions		3 (25%)			2 (100%)		5 (21%)
	Other (non‐profit organisation, charitable foundation, independent research and consultancy)	1 (50%)	1 (8%)		1 (25%)			2 (8%)
	None	1 (50%)	6 (50%)	1 (100%)	1 (25%)		4 (80%)	12 (50%)
	Not reported		3 (25%)		2 (50%)		1 (20%)	6 (25%)

^a^ Systematic scoping review (*n* = 1), rapid SR (*n* = 1), rapid systematic review meta‐analysis (*n* = 1), quantitative evidence synthesis (*n* = 1), and mini‐review (*n* = 1).

^b^ Denominator is 20 as meta‐analysis is generally not part of ScR methodology.

### 3.1 Study characteristics

All syntheses were published in English and historical and new syntheses had a median of 4.5 (range 1–7) and 6.5 (range 1–18) authors, respectively (Table 1). The historical syntheses were peer‐reviewed articles; one published in October 2019 and one in February 2022. Among the new syntheses, 50% (12/24) were peer‐reviewed published articles, 8% (2/24) were short communication products, 4% (1/24) were letters to the editor, 4% (1/24) commentaries, and 33% (8/24) were originally posted as preprints, of which 4 were published as of December 2022. Historical and new syntheses focused on the general population (100%; 2/2 and 92%; 22/24). Funding sources were reported in 77% (20/26). Meta‐analysis was conducted in 50% (11/22) of applicable syntheses, one of which was historical.

The median number of databases searched was 3 (range 2–4) in historical and 4 (range 1–8) in new syntheses. There was no notable difference by review type, suggesting the variability did not depend on type of review. PubMed was the most widely searched database (81%; 21/26), followed by Scopus (58%; 15/26) and Embase (54%; 14/26). None of the historical and 21% (5/24) of new syntheses searched preprint databases, with medRxiv being the most utilized (13%; 3/24). A grey literature search was conducted in historical and new syntheses (100%; 2/2 and 25%; 6/24). Most syntheses (69%; 18/26) also implemented search verification strategies, with reference list checking being the most widely used (58%; 15/26). None of the historical syntheses and 46% (11/24) of the new syntheses were mislabeled based on authors' reported methods. Reviewers determined that one labelled RR was in fact a SR, one ScR was a RR, four SRs were RRs, and of the five “other” syntheses that used unconventional labels, two were SRs and three were RRs.

### 3.2 Utility

Historical syntheses included a median of 69 (range 66–71) studies while new syntheses included median 15 (range 4–77), of which a median of 8 (range 0–19) studies were published/posted after May 1, 2022 (Table 2). This suggests new syntheses included fewer studies from the 2022 outbreak compared to historical syntheses while some new syntheses (8%; 2/24) did not include any new evidence from the 2022 outbreak. A third (33%; 8/24) of new syntheses included only new evidence and 58% (14/24) included new and historic evidence. However, only 29% (7/24) contrasted findings between new and historical evidence. One of these syntheses compared two historic articles that were not included in their study [17]. New syntheses were conducted in a shorter time frame than historical syntheses. The median time for the peer‐review process, measured as submission to first publication date was 33 (range 32–33) weeks and 7 (range 1–16) weeks for historical and new syntheses, respectively. A measure of how up to date the review was at first availability was measured as median time from last search date to first publication/preprint post date was 68 (range 61–75) weeks and 6 (range 1–20) weeks for historical and new syntheses, respectively. The four syntheses that were originally posted as preprints before being published were available a median of 3 (range 1–6) weeks after their last search date, whereas the published versions were not available for a median 11 (range 1–14) weeks, which is 8 weeks after the preprints were posted (File S2).

**Table 2:** Utility assessment of evidence syntheses.

Characteristic	Categorization	Systematic review ± meta‐analysis		Meta‐analysis (*N* = 1)	Scoping review (*N* = 4)	Rapid review (*N* = 2)	Other^a^ (*N* = 5)	Total new synthesis (*N* = 24)
		Historical (*N* = 2)	2022 outbreak (*N* = 12)					
Median no. of included studies in syntheses (range)	Total	69 (66–71)	15 (4–46)	8 (8–8)	13 (5–77)	37 (12–62)	15 (12–21)	15 (4–77)
	Published/posted preoutbreak	69 (66–71)	2 (0–17)		3 (0–74)	28 (12–43)	6 (0–12)	4 (0–74)
	Published/posted during outbreak		8 (0–43)	8 (8–8)	7 (1–15)	10 (0–19)	8 (7–15)	8 (0–19)
No. of syntheses that included evidence pre‐outbreak, during outbreak or both (*n*, %)	Only pre‐outbreak studies	2 (100%)	1 (8%)			1 (50%)		2 (8%)
	Only during outbreak studies		5 (42%)	1 (100%)	1 (25%)		1 (20%)	8 (33%)
	Both pre‐ and during outbreak studies		6 (50%)		3 (75%)	1 (50%)	4 (80%)	14 (58%)
Comparison/contrast between new and historical evidence (*n*, %)	Yes		3 (25%)			1 (50%)	3 (60%)	7 (29%)
	No		6 (50%)	1 (100%)	3 (75%)		2 (40%)	12 (50%)
	No historic/new evidence to contrast among new syntheses		3 (25%)		1 (25%)	1 (50%)		5 (21%)
Limitations of the evidence reported (*n*, %)	Quality of studies	1 (50%)	2 (17%)		1 (25%)		1 (20%)	4 (17%)
	Limited number of studies	1 (50%)	6 (50%)		2 (50%)			8 (33%)
	Limited number of participants	1 (50%)	5 (42%)		1 (25%)	1 (50%)	1 (20%)	8 (33%)
	Strength of conclusions/evidence	2 (100%)	6 (50%)		2 (50%)	1 (50%)	2 (40%)	11 (46%)
	Lack of recent data	1 (50%)					1 (20%)	1 (4%)
	Limitations of review process				2 (50%)			2 (8%)
	Other		2 (17%)		1 (25%)	1 (50%)	1 (20%)	5 (21%)
	No/Not reported		3 (25%)	1 (100%)			2 (40%)	6 (25%)
Identified if there were previously published evidence synthesis (*n*, %)	Yes (identified other SRs)	1 (50%)			1 (25%)			1 (4%)
	No (identified this as the first)	1 (50%)	6 (50%)	1 (100%)		1 (50%)	3 (60%)	11 (46%)
	Not reported (did not identify any)		6 (50%)		3 (75%)	1 (50%)	2 (40%)	12 (50%)
Published syntheses previously posted as a preprint (*n*, %)	Yes		3 (25%)				1 (20%)	4 (17%)
	No	2 (100%)	7 (58%)	1 (100%)	3 (75%)	2 (100%)	3 (60%)	16 (67%)
	N/A – preprint that was not published		2 (8%)		1 (25%)		1 (20%)	4 (33%)
Timeliness median (range)	Weeks between submission & publication date (Total no. syntheses)	33 (32–33) 2	6 (2–16) 7	2 (2–2) 1	8 (7–8) 2	4 (1–7) 2	7 (4–11) 3	7 (1–16) 15
	Weeks between preprint to publication date (Total no. syntheses)		8 (5–9) 3				12 (12–12) 1	9 (5–12) 4
	Weeks between last search date to publication date (Total no. syntheses)	68 (61–75) 2	10 (2–17) 7	2 (2–2) 1	20 (8–20) 3	12 (6–17) 2	16 (1–16) 3	10 (1–20) 16
	Weeks between last search date to preprint post date (Total no. syntheses)		4 (1–15) 5		2 (2–2) 1		7 (1–14) 2	3 (1–15) 8
	Weeks between last search date to first publication/preprint post date (Total no. syntheses)	68 (61–75) 2	6 (1–17) 12	2 (2–2) 1	14 (2–20) 4	12 (6–17) 2	14 (1–16) 5	6 (1–20) 24

^a^ Other synthesis as reported by review authors included a systematic ScR (*n* = 1), rapid SR (*n* = 1), rapid SR‐MA (*n* = 1), quantitative evidence synthesis (*n* = 1) and mini‐review (*n* = 1).

**Figure 2:**
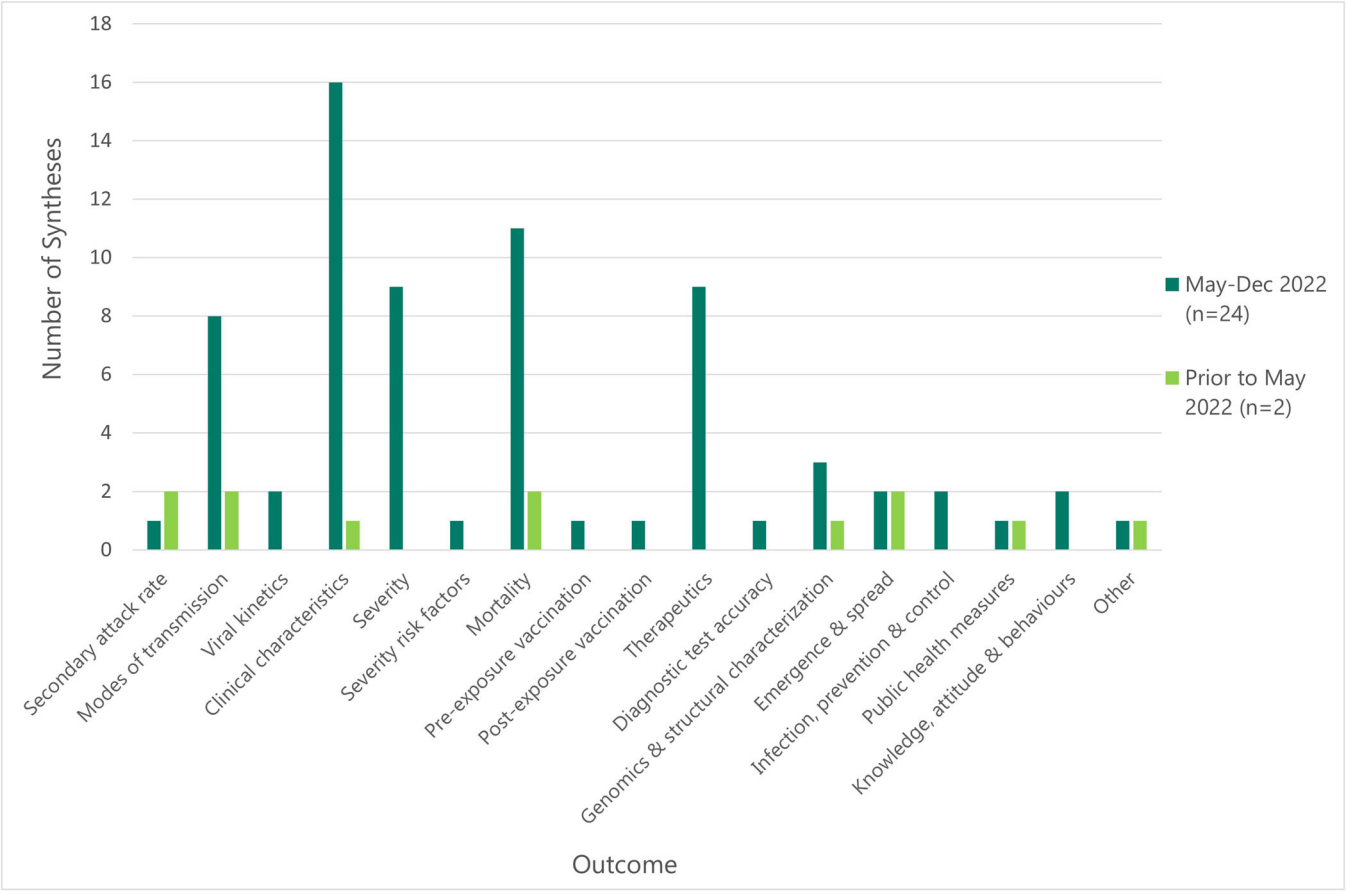
Total number of syntheses per outcome or foci reported. Other: Miscarriage, intrauterine demise, neonatal death, overall fetal or neonatal losses, alive, fetal anomalies, preterm birth <37 weeks, and epidemiology.

The research questions were broad in historical syntheses and aimed to describe the evolution and epidemiology of mpox over time (e.g., secondary attack rate and modes of transmission) (Figure 2). In comparison, most new syntheses had a narrow research question, which may explain why they had fewer included studies (Table 2). There was significant overlap among new syntheses for a number of topic areas (File S3) and outcomes such as clinical characteristics (67%; 16/24), mortality (46%;11/24) (Figure 2). Half (12/24) of new syntheses either did not identify whether a previously published synthesis was available on the topic/outcome or authors stated they were the first to describe the topic/outcome (46%; 11/24), but this was the case for only 36% (4/11) of these syntheses (Table 2).

**Figure 3:**
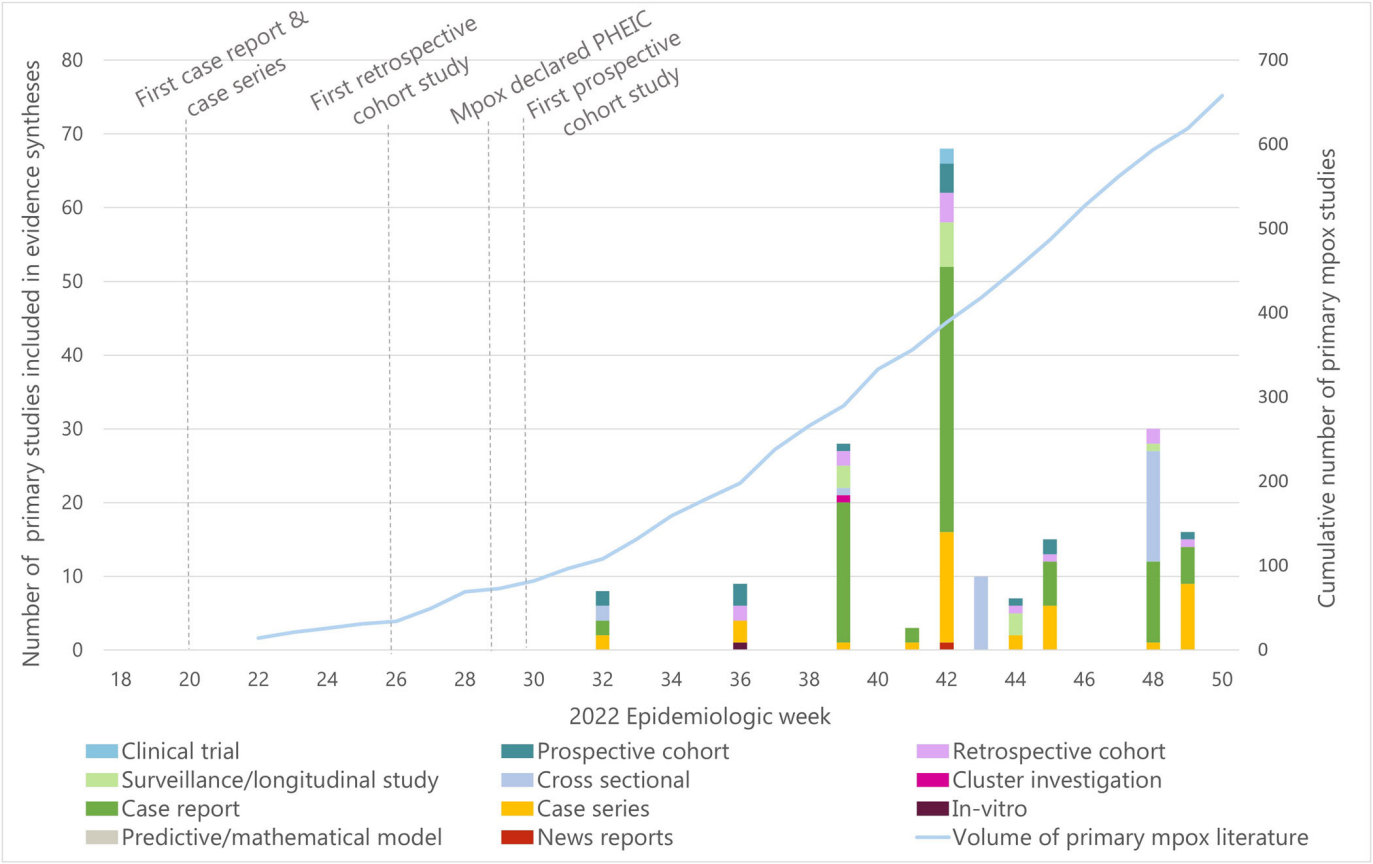
Evidence syntheses that reported study designs of new evidence included from the 2022 outbreak (*N* = 18) by publication date (May to December 2022) stacked by study design synthesis where the search date was a median of 6 weeks before publication/preprint post date. The volume of new primary research on mpox is the line graph over the same period.

Study designs included in the syntheses were poorly described and often incorrectly labelled or were not reported in one historical SR and four new syntheses), making it difficult to compare across syntheses summarizing the same outcome (File S4). Two new syntheses also did not specify whether the included study designs were published before or during the 2022 outbreak. Early publications in the 2022 mpox outbreak were mostly descriptive studies (Figure 3). Among all syntheses that reported study designs (*n* = 21), case reports (71%; 15/21) and case series (86%; 18/21) were the most common study design with a median of 2 (range 0–31) and 2 (range 1–10) included, respectively (File S4). The first analytical studies (i.e., cohort studies) started to frequently appear 16 weeks into the outbreak (August 14–20, 2022), which was after the search date of 58% (14/24) of the syntheses. As a result, only 33% (8/24) of new syntheses included prospective cohorts, 38% (9/24) retrospective cohorts, and 38% (9/24) surveillance data analysis with a median of 1.5 (range 1–3), 1 (range 1–2), and 3 (range 0–6) studies, respectively (Figure 3, File S4). In comparison, one historical SR included analytical observational studies such as surveillance data analyses (*n* = 43) while a smaller proportion were cluster investigations (*n* = 11), case reports (*n* = 9), and case series (*n* = 1) [4] and the other did not specify [5].

Limitations varied across syntheses and were not reported in 24% (6/24) of new syntheses despite being a criteria in the PRISMA guidelines (Table 2). Lack of confidence in the conclusions and evidence was the most common limitation reported in historical (100%; 2/2) and among the 18 new syntheses that reported limitations (61%; 11/18).

### 3.3 Methodological rigor and quality

Most new syntheses used at least two reviewers to conduct relevance screeningand data extraction (67%; 16/24), while the remaining did not report details (Table 3). One historical SR did not report details on screening and extraction while the other conducted 10% in duplicate and the remaining by a single reviewer. One historical SR did not report using a validated ROB tool and narratively described bias [4] while the other reported that ROB was not conducted because there were no tools available for the included study designs [5]. ROB was performed in 55% (11/20) of new syntheses, excluding the ScRs, and varied across review types (Table 3). Among syntheses that conducted ROB, duplicate reviewers were frequently used to evaluate ROB in 73% (8/11) of syntheses, while 19% (3/11) did not report the number of reviewers. Five different ROB tools were used across syntheses and in 27% (3/11) the tools were modified by authors to create an overall score (1/3) or to remove items that were NA (2/3). Overall ROB was described in the results of 91% (10/11) of these syntheses, as well as one ScR that did not conduct a critical appraisal. None of the 26 syntheses evaluated the certainty of evidence using a validated framework such as Grading of Recommendations, Assessment, Development, and Evaluations (GRADE) or reported including an experienced methodologist (Table 3) [18].

**Table 3:** Methodological rigor and certainty of evidence syntheses.

Characteristic	Categorization	Systematic review ± meta‐analysis		Meta‐analysis (*N* = 1)	Scoping review (*N* = 4)	Rapid review (*N* = 2)	Other^a^ (*N* = 5)	Total new syntheses (*N* = 24)
		Historical (*N* = 2)	2022 outbreak (*N* = 12)					
No. of reviewers that assessed study screening	1							
	2+		11 (92%)		2 (50%)	1 (50%)	2 (40%)	16 (67%)
	Not reported	1 (50%)	1 (8%)	1 (100%)	2 (50%)	1 (50%)	3 (60%)	8 (33%)
	Other (10% full text evaluated in duplicate and remaining 90% by one reviewer)	1 (50%)						
No. of reviewers that assessed study extraction	1							
	2+		9 (75%)		3 (75%)	1 (50%)	3 (60%)	16 (67%)
	Not reported	1 (50%)	3 (25%)	1 (100%)	1 (25%)	1 (50%)	2 (40%)	8 (339%)
	Other (one reviewer screened and a second reviewer randomly checked 10% of articles)	1 (50%)						
No. of reviewers that conducted risk of bias	1							
	2		5 (42%)			1 (50%)	2 (40%)	8 (40%)^b^
	Not reported		3 (25%)					3 (15%)^b^
	Risk of bias not conducted	2 (100%)	4 (33%)	1 (100%)		1 (50%)	3 (60%)	9 (45%)^b^
	Not part of methodology				4 (100%)			N/A
Risk of bias conducted	Yes—risk of bias conducted and tools reported		7 (58%)			1 (50%)	2 (40%)	10 (50%)^b^
	Newcastle Ottawa Scale^c^		4 (33%)				1 (20%)	5 (25%)^b^
	Joanna‐Briggs critical appraisal tool^c^		2 (17%)				1 (20%)	3 (15%)^b^
	National Heart, Lung, and Blood Institute (NIH) QA tool^c^		2 (17%)				1 (20%)	3 (15%)^b^
	Tool developed by Murad et al. for case reports/series^c^		1 (8%)			1 (50%)	1 (20%)	3 (15%)^b^
	Risk of bias not assessed using a validated tool	1 (50%)	1 (8%)					1 (5%)^b^
	No—risk of bias not conducted	1 (50%)	4 (33%)	1 (100%)		1 (50%)	3 (40%)	9 (45%)^b^
	Not part of methodology				4 (100%)			N/A
Evaluated certainty of evidence (GRADE)	Yes							
	No	2 (100%)	12 (100%)	1 (100%)		2 (100%)	5 (100%)	20 (100%)^b^
	Not part of methodology				4 (100%)			N/A
Methodologist included in conduct of synthesis	Yes							
	No	2 (100%)	12 (100%)	1 (100%)	4 (100%)	2 (100%)	5 (100%)	24 (100%)

^a^ Systematic scoping review (*n* = 1), rapid SR (*n* = 1), rapid systematic review meta‐analysis (*n* = 1), quantitative evidence synthesis (*n* = 1) and mini‐review (*n* = 1).

^b^ Denominator is 20 as risk of bias is generally not part of ScR methodology.

^c^ Sum of articles across tools can be greater than the total articles as more than one tools can be used by an evidence synthesis.

**Figure 4:**
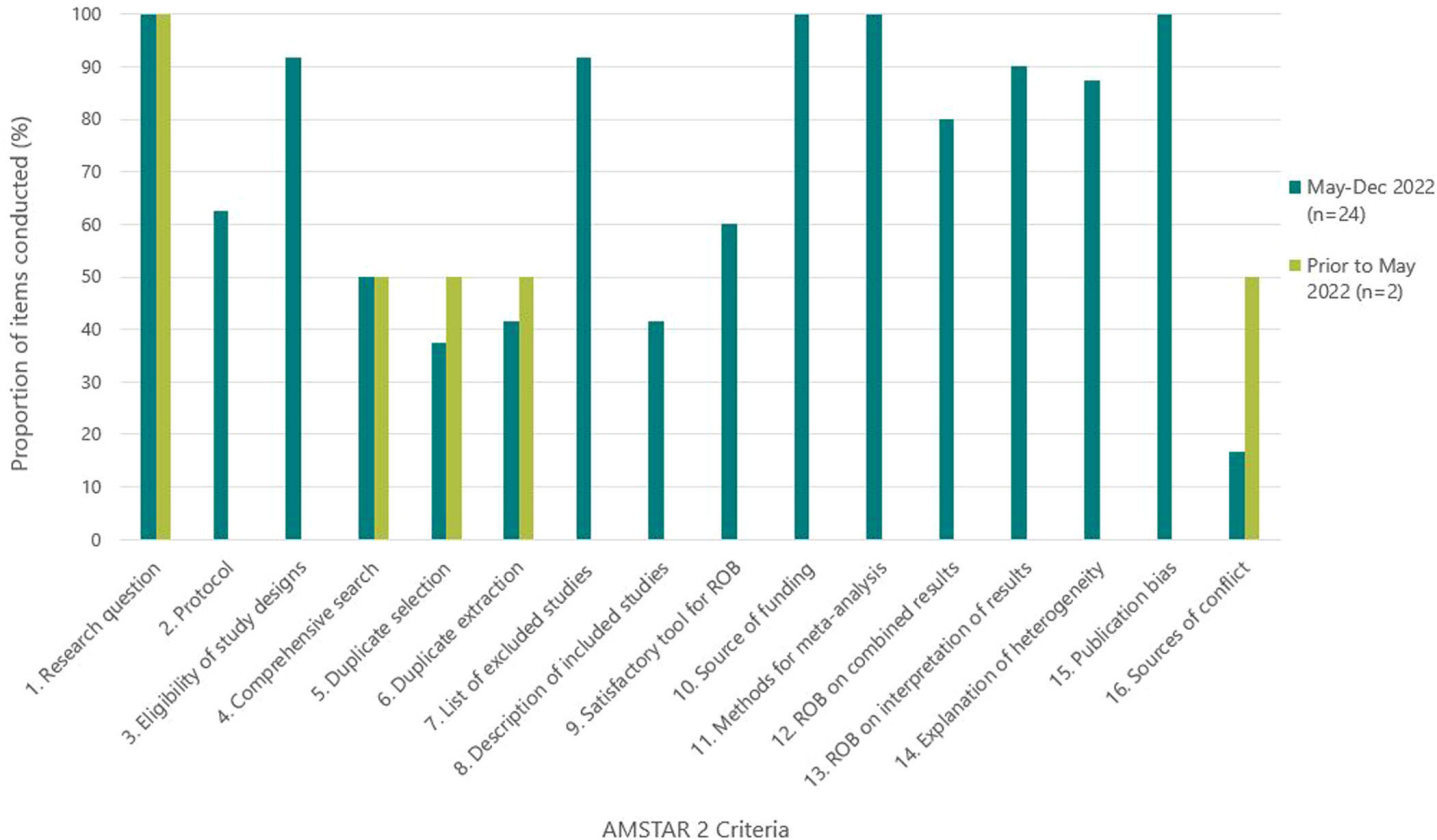
Proportion of AMSTAR‐2 items that were adequately conducted by the included evidence syntheses (*N* = 26) included in this evaluation.

The methodological quality of historical syntheses (100%; 2/2) were rated as critically low and the new syntheses were low (8%; 2/24) and critically low (92%; 22/24) (File S5). All syntheses provided a research question and inclusion criteria, most provided a conflict of interest statement, and performed selection and extraction with at least two reviewers (Figure 4 and File S4). However, conduct was poor among critical AMSTAR‐2 items in all syntheses. Most did not provide a protocol, provide a list of excluded studies, or account for ROB when interpreting results, and none used an appropriate statistical method to combine results. The overall quality rating of the four syntheses that were originally posted as preprints were the same after being published, however, the methodological conduct of four items improved in two syntheses (File S2).

### 3.4 Reporting quality

**Figure 5:**
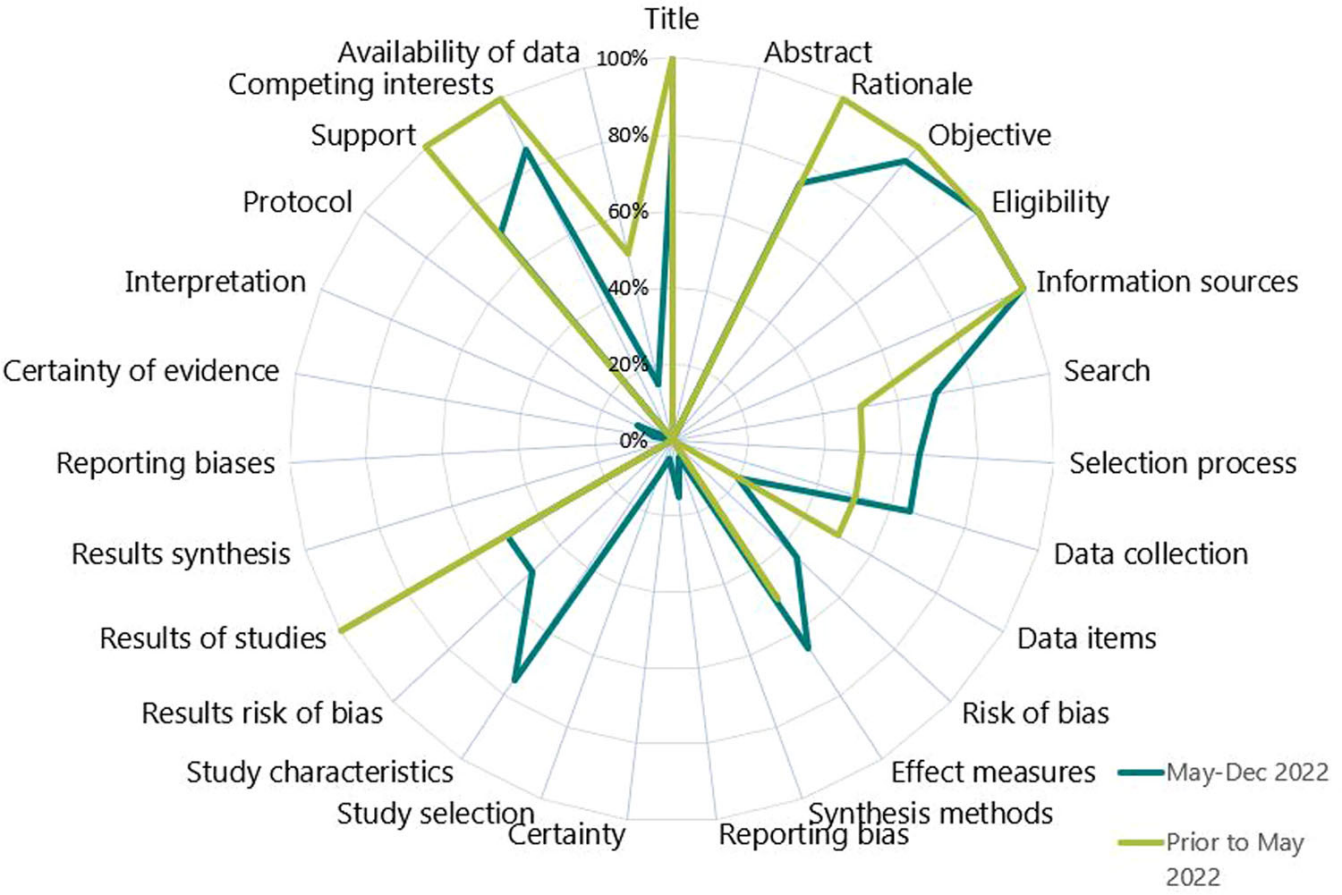
Proportion of evidence syntheses (*N* = 22) that met the items in the PRISMA 2020 checklist.

**Figure 6:**
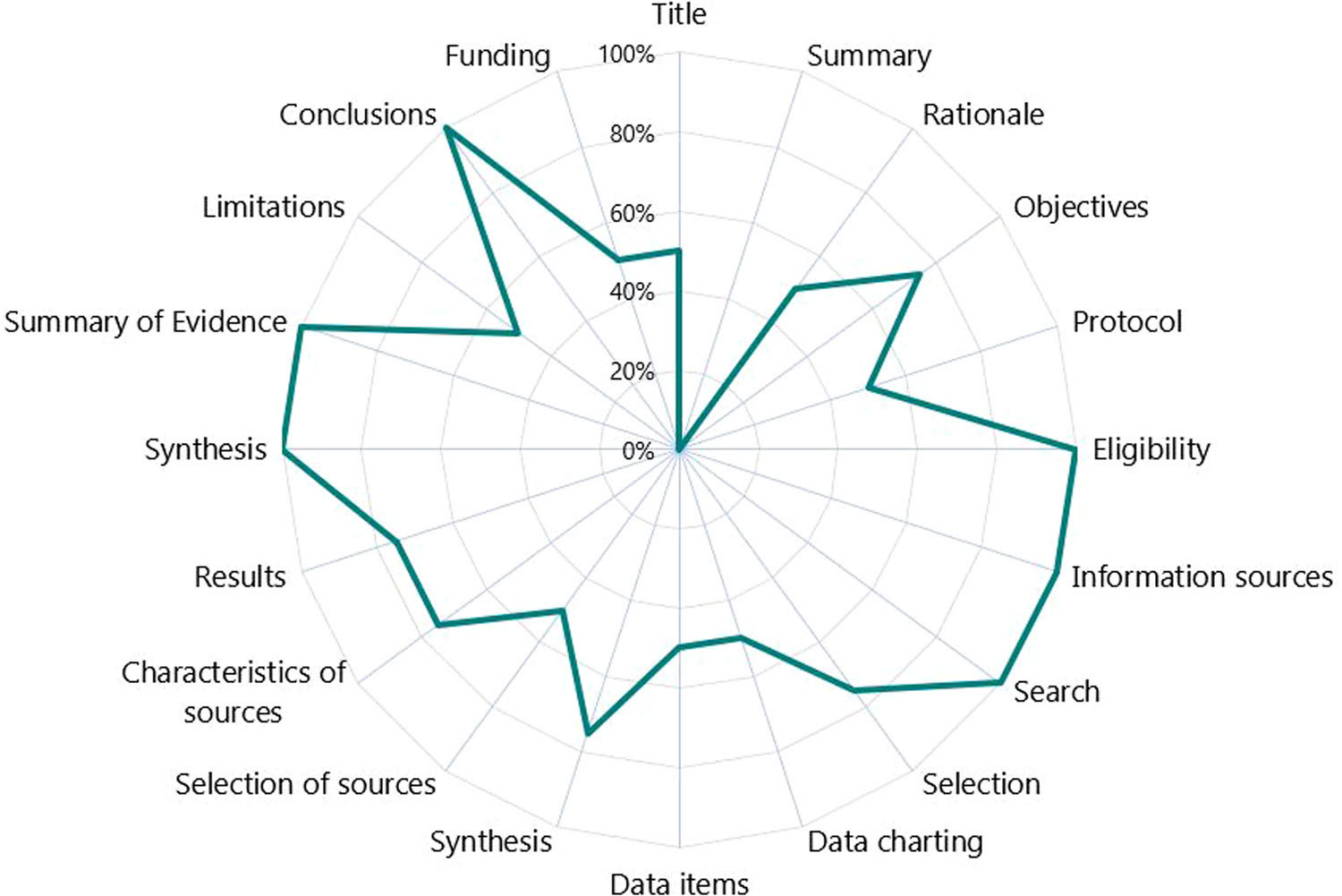
Proportion of scoping reviews from May to December 2022 (*N* = 4) that met the items in the PRISMA‐ScR checklist.

The reporting quality of 26 evidence syntheses were assessed using PRISMA 2020 (*n* = 22), PRISMA abstracts (*n* = 23), and PRISMA‐ScR (*n* = 4) checklists (File [Link], [Link]). Of the 27 items in the PRISMA 2020 checklist, a median of 10.5 (range 10–11) items and 11.5 (range 4–21) items out of 27 items were sufficiently reported by historical and new syntheses, respectively. The median number of items reported increased slightly for the four preprints that were later published from median 14.5 (range 10–22) to 15.5 (range 14–22) items, respectively (File S2). Title, objective, eligibility criteria, information on sources, and competing interests were reported in more than 80% of all syntheses (Figure 5). Three additional items (i.e., rationale, results of individual studies, and financial/nonfinancial support) were reported in both historical syntheses. The abstract, results of syntheses, reporting biases, and protocol registration were poorly reported among all synthesis types. The four new ScRs evaluated using PRISMA‐ScR had a median of 14 (range 9–18) out of 22 items that were sufficiently reported. Eligibility criteria, information on sources, search, synthesis, summary of evidence, and conclusions were reported in more than 80% of syntheses (Figure 6). All ScRs lacked a structured summary.

**Figure 7:**
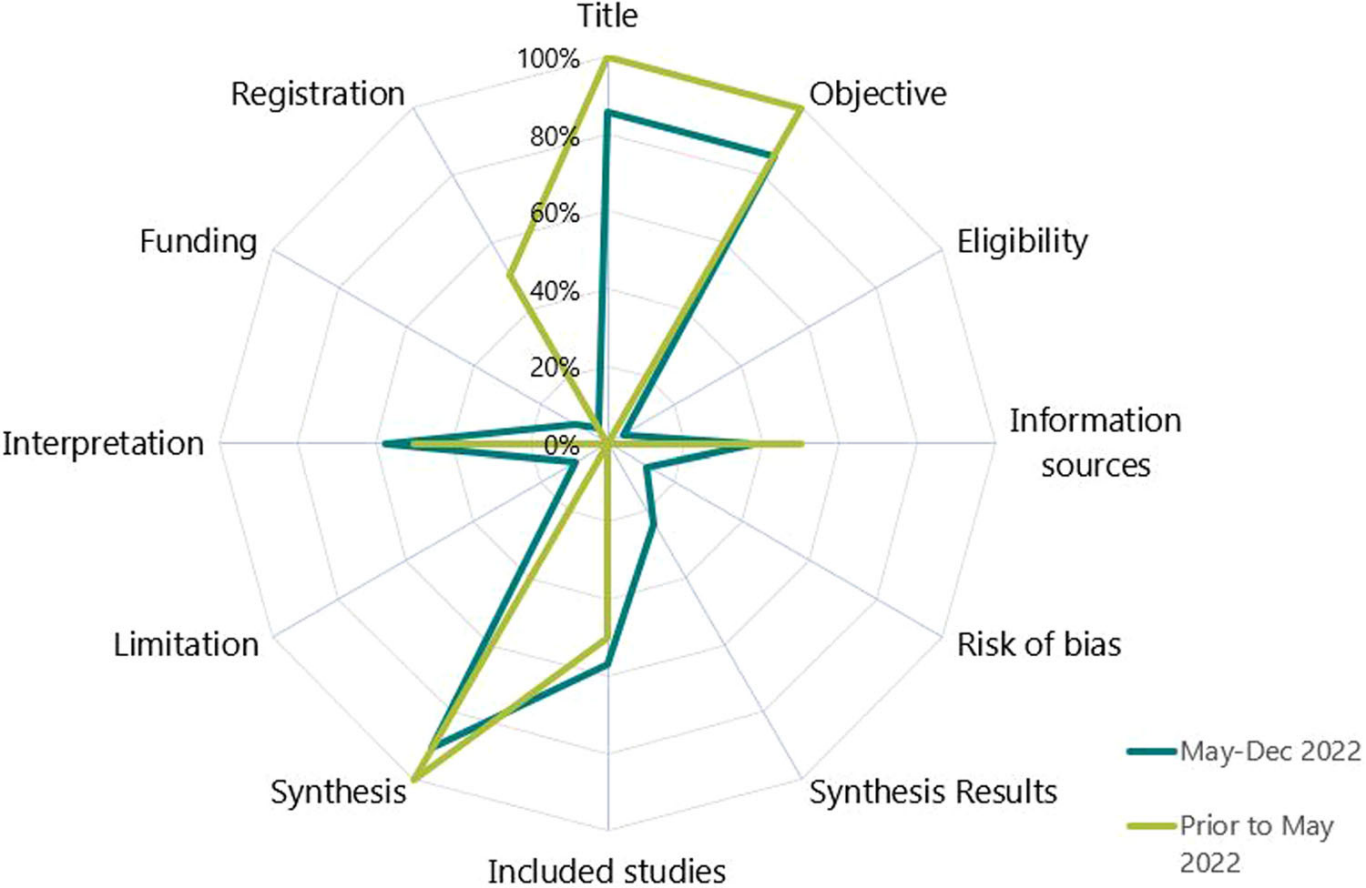
Proportion of evidence synthesis abstracts (*N* = 22) that met the items in the PRISMA abstracts checklist.

Three evidence syntheses did not include an abstract: one peer‐reviewed article, one letter to the editor, and one commentary/correspondence [19, 20, 21]. Of the 12 items in the PRISMA‐abstracts extension, a median of 5 (range 4–6) and 5 [1, 2, 3, 4, 5, 6, 7, 8, 9] items were adequately reported by historical and new syntheses, respectively. The median number of items reported increased slightly for the four preprints that were later published from median 6.5 (range 4–9) to 7 (range 4–10) items, respectively (File S2). Title, objective, and synthesis of main outcomes were reported in over 80% of historical and new syntheses (Figure 7). Abstracts generally lacked information on the eligibility criteria, risk of bias/critical appraisal, synthesis of results, limitations, and funding.

## 4 DISCUSSION

New evidence accrues rapidly during a public health emergency and there is an urgent need by decision‐makers to stay on top of the continuously shifting, and in some cases contradictory evidence [10]. Timely synthesis of evidence during outbreak events is valued by end‐users as a source of unbiased, reliable, and accurate information to support evidence‐based decision‐making [22, 23]. Typically evidence syntheses can take 8 to 20 months to produce and publish [24, 25]. Using the 2022 mpox outbreak as an example, the utility and quality of evidence syntheses produced at a much faster pace during a public health emergency were evaluated to explore the adherence to the proper methods and reporting, and to explore the utility of those syntheses for decision‐making considering the rapidly evolving evidence.

Decisions are made quickly during a public health emergency and are ideally based on the available body of evidence, which is most useful and digestible when synthesized, and at most a few days out‐of‐date. The mpox evidence syntheses produced in the first 6 months of the outbreak took a median of 10 weeks from the last search date to publication date and the 33% that were preprints were posted a median of 3 weeks after the last search. For evidence syntheses this is an accelerated review cycle, but during a fast‐moving public health event these syntheses were considered out‐of‐date at time of publication, providing minimal value for public health decision‐making. Decision‐makers were left to digest individual primary studies without having an evidence synthesis to support understanding each study in the context of the body of evidence as a whole [10].

Given that it takes time to design and conduct analytical studies, new syntheses incorporated mostly descriptive studies, which by the nature of their design are at high risk of bias as they are missing a comparator group and are subject to missing information, selection bias, and confounding. Thus, the evidence from these syntheses is considered low/very low quality and provide a low level of certainty in the findings. The findings from early outbreak syntheses are likely to change quickly as new evidence is published including retrospective and prospective cohorts, which began to emerge 11 and 16 weeks into the outbreak, respectively.

There were two observations of major duplication across the mpox syntheses: first, many new syntheses were redundant as they overlapped in their topic areas and outcomes. Evidence from some new syntheses were also covered by historical SRs as they were conducted when there was minimal new data from the 2022 outbreak. Second, many new syntheses did not identify previously published syntheses before conducting an evidence synthesis, thereby contributing to research waste. Evidence from the 2022 outbreak was often not compared with historical evidence to identify alignment or deviations of previous findings with the current outbreak, which was a key question that public health decision‐makers wanted addressed. The duplication may have been due in part to syntheses projects occurring simultaneously, but there was also a lack of due diligence to justify the added value of the conducted syntheses. These observations support the growing concern from synthesis experts about the extent of research waste and for further examination of how evidence synthesis can support decision‐making during public health emergencies [26, 27].

Syntheses have been scrutinized for their variability in methodological rigor and reporting, although tools are readily available to guide review authors through proper conduct and reporting [14, 28]. This study and others have found that despite the availability of these tools, evidence syntheses during emergencies have been poorly conducted and many required reporting items are frequently omitted [13, 29, 30]. These deficiencies were evident even in historical SRs, suggesting that researcher education and improved peer‐review are needed to improve methodological rigor and reporting. Omission of ROB within evidence syntheses was often not explained [5]. While there are checklists available for most study designs, it can be difficult to know which are validated and acceptable to use. This highlights the need to involve an experienced methodologist in synthesis research to ensure proper conduct with the most appropriate tools [31, 32]. The methodological rigor of many identified evidence syntheses was substandard, which undermines the transparency, trustworthiness, and ultimately the utility of syntheses to aid in decision‐making.

A living evidence approach where synthesized information would be at most a few days out‐of‐date can alleviate the issues that are described in this study. Types of living products include living SRs or living evidence profiles where evidence can be systematically identified, mapped, synthesized, and disseminated in a rapid on‐going cycle for decision‐makers to quickly identify new information in the context of what is already known as well as identify knowledge gaps [33, 34, 35]. Guidance for living evidence syntheses are actively being produced which will provide methods to improve the quality and utility of evidence syntheses during a public health event such as mpox [36, 37].

## 5 LIMITATIONS

Some evidence syntheses could have been missed due to search terms being limited to English and French, failure to publish, and/or lack of citation indexing. A limitation of using single reporting quality scores is that the differential impact of the missing reporting items and how these impact our understanding of how a study is conduct is not captured in a score. This was the first study to evaluate utility of evidence syntheses during the first 6 months of an emergent outbreak. The criteria was developed by our team based on criteria used to decide when to conduct a new or updated evidence synthesis and our experience in conducting rapid evidence syntheses during emergent public health outbreaks [2, 31, 36]. Further evaluation of these criteria is warranted. Readers are cautioned that findings of this research only apply to the first few months of an outbreak, when evidence is changing rapidly, and may not apply to evidence syntheses produced at later periods of the outbreak.

## 6 CONCLUSIONS

This is the first study to systematically evaluate the utility and quality of evidence syntheses produced during the first 6 months of a public health emergency and provide comparisons to syntheses published before the emergency. The results suggest that production and publication lag times during an event when evidence accrues rapidly results in syntheses that are often outdated by the time of publication. The utility of these syntheses are hampered by a plethora of methodological and reporting issues, resulting in poor‐quality syntheses that are unreliable for decision‐making. The overlapping content across evidence syntheses and lack of new analytical evidence meant that new syntheses added minimal value to the existing literature, contributing to research waste. This study highlights that syntheses need better adherence to validated guidelines, researchers should make better use of existing syntheses, and quality assessment tools are needed to evaluate the types of studies produced at the beginning of an outbreak. Mechanisms to produce and disseminate continuously updated living evidence synthesis products need to be explored to support decision‐makers responding to a public health emergency.

## AUTHOR CONTRIBUTIONS

**Kusala Pussegoda**: Conceptualization; data curation; formal analysis; investigation; methodology; project administration; validation; visualization; writing—original draft; writing—review and editing. **Izza Israr**: Conceptualization; data curation; formal analysis; methodology; project administration; writing—original draft; writing—review and editing. **Austyn Baumeister**: Data curation; methodology; writing—review and editing. **Tricia Corrin**: Data curation; writing—review and editing. **Melanie Sterian**: Data curation; writing—review and editing. **Mavra Qamar**: Data curation; writing—review and editing. **Anmol Samra**: Data curation; writing—review and editing. **Lisa Waddell**: Conceptualization; methodology; project administration; supervision; writing—original draft; writing—review and editing.

## CONFLICT OF INTEREST STATEMENT

The authors declare no conflict of interest.

## PEER REVIEW

The peer review history for this article is available at https://www.webofscience.com/api/gateway/wos/peer-review/10.1002/cesm.70005.

## Supporting information

Supporting information.

Supporting information.

Supporting information.

Supporting information.

Supporting information.

Supporting information.

Supporting information.

Supporting information.

## Data Availability

The data that support the findings of this study are openly available in Open Science Framework at https://osf.io/4MEA6/.
