# Supplementary material for: Methodological and reporting quality of systematic and rapid reviews on human mpox and their utility during a public health emergency
Source: Cochrane Evid Synth Methods. 2024 Nov 15;2(11):e70005. doi: 10.1002/cesm.70005 (PMC11795912; doi:10.1002/cesm.70005)
Supplement: Supplementary file 1 — Supporting information. [file CESM-2-e70005-s008.docx]

Methodological and reporting quality of systematic and rapid reviews on human mpox and their utility during a public health emergency: Protocol

Authors: Izza Israr ^1,2^, Kusala Pussegoda ^1^, Tricia Corrin ^1^, Austyn Baumeister ^1^, Anmol Samra^1,2^, Melanie Katz ^1^, Mavra Qamar ^1^, Lisa Waddell ^1c^

^1^Public Health Risk Sciences Division of the National Microbiology Laboratory, Guelph ON, Public Health Agency of Canada.

^2^University of Guelph, Department of Population Medicine

Important Dates

Evidence published up to December 31, 2022

Protocol version 1 initiated on May 24, 2023

Protocol version 2 initiated on July 27, 2023 to reflect changes made after piloting data extraction form

Protocol version 3 initiated on September 26, 2023 to reflect changes made prior to analysis of data

Table of Contents

[Rationale 3](#_Toc172803621)

[Background 3](#_Toc172803622)

[Objective 4](#_Toc172803623)

[Research questions 4](#_Toc172803624)

[Planned study outputs 4](#_Toc172803625)

[Methods 4](#_Toc172803626)

[Eligibility criteria 4](#_Toc172803627)

[Inclusion Criteria 4](#_Toc172803628)

[Exclusion criteria 5](#_Toc172803629)

[Information sources and search strategy 5](#_Toc172803630)

[Grey literature 6](#_Toc172803631)

[Study selection 6](#_Toc172803632)

[Review management 6](#_Toc172803633)

[Article screening 6](#_Toc172803634)

[Article characterization 7](#_Toc172803635)

[Quality and reporting 7](#_Toc172803636)

[Data synthesis 8](#_Toc172803637)

[Appendix 1. Search strategies for mpox studies published during the 2022 outbreak 9](#_Toc172803638)

[Appendix 2. Search strategies for mpox evidence syntheses published prior to the 2022 outbreak 10](#_Toc172803639)

[Appendix 3. Relevance screening form 11](#_Toc172803640)

[Appendix 4. Data characterization form 13](#_Toc172803641)

[References: 47](#_Toc172803643)

## Rationale

The methodological and reporting quality of evidence syntheses are of interest to public health decision makers who use evidence in decision-making for routine work or during public health emergency. If evidence syntheses are to be used for developing recommendations and identifying knowledge gaps the methodology must remain rigourous in the collation, appraisal and synthesis of the evidence regardless of the driver for producing the evidence synthesis. The rigor and reliability of these reviews are closely associated with the methodological and reporting quality and end-users should seek high quality evidence syntheses for decision-making. Using the mpox outbreak that began in May 2022, this study will evaluate the quality of conduct and reporting of new vs. Existing evidence syntheses to explore the utility and drivers of conducting conventional evidence synthesis during a public health emergency, particularly at the beginning of an outbreak when there is limited new evidence available and recent pre-outbreak evidence syntheses exist.

## Background

On July 23, 2022, the World Health Organization (WHO) declared mpox (formerly known as monkeypox) a Public Health Emergency of International Concern (PHEIC) (1, 2). As of June 5, 2023 there have been 87,929 confirmed cases and 146 deaths reported to the WHO from 110 member states(1). Since the global weekly case peak of 7,576 the week of August 8, 2022 there has been a steady decline in weekly cases reported(1). Mpox is a zoonotic disease caused by an orthopoxvirus in the poxviridae family (1, 2). Mpox was initially discovered in the Democratic Republic of Congo in 1970, and has largely been contained to central Africa with occasional cases reported in non-endemic countries (3, 4). Mpox presents as a mild to severe disease in humans and typically includes a pox rash, lymphadenopathy that can cause severe pain and discomfort, fever, malaise, and other symptoms that may lead to a variety of complications(1, 2). Transmission of the mpox virus from animal-to-human and human-to-human occurs mainly through direct close contact with skin lesions, body fluids or respiratory droplets, but can also transmit indirectly via contaminated materials such as bedding or clothes(1, 2, 5).

Evidence syntheses such as systematic reviews, rapid reviews and scoping reviews aim to identify relevant studies, critically assess and synthesize information to comprehensively evaluate the findings on a topic which are utilized by end-users for public health decision-making. Prior to the 2022 mpox outbreak, recent systematic reviews using rigorous systematic review methodologies had summarized the primary literature on mpox with evidence up to 2020 (6, 7). Despite the lack of new high quality studies reporting evidence on the 2022 mpox outbreak there was an increase in evidence syntheses in the first 6 months of the outbreak that were produced in a short time frame which may have sacrificed methodological rigor and quality. Studies have shown that during times of a high impact event there is rapid evidence accrual, and evidence syntheses such as systematic reviews tend to be produced and published in shortened timelines (8). Although more timely, the methodological rigour (9), reporting (10), and utility of evidence syntheses to guide decision-making during an event need to be evaluated for how quickly they become outdated, or irrelevant with the rapid emergence of new primary evidence (11, 12). To date, the methodological and reporting quality of mpox evidence synthesis of human studies as well as their utility during the outbreak is unknown.

## Objective

The objective of this study is to evaluate the utility and adherence of evidence syntheses produced during the first six months of the mpox outbreak to the A Measurement Tool to Assess Systematic Reviews (AMSTAR) quality assessment tool and Preferred Reporting Items for Systematic Reviews and Meta-Analyses (PRISMA) or appropriate extension guidelines using the 2022 mpox outbreak as an example. Evidence syntheses on human mpox studies available prior to the outbreak will be contrasted with evidence syntheses produced during the first six months of the outbreak to examine adherence to methodological quality and reporting tools and drivers for conducting the syntheses (e.g. topic areas and outcomes covered) so we can evaluate the utility or duplication of synthesis efforts at the beginning of an outbreak.

### Research questions

**Key Question 1)** What is the methodological and reporting quality of the evidence syntheses conducted during the first six months of the 2022 mpox outbreak and how does it compare to evidence syntheses conducted prior to the outbreak?

**Key Question 2)** What was the purpose and utility of conducting evidence syntheses during the first six months of the 2022 mpox outbreak?

### Planned study outputs

1. Methodological study

## Methods

This study has been developed and conducted by individuals with methodological expertise in evidence synthesis. KP and II will be tasked with leading the study including the development of the protocol, conduct and writing of the methodological review. LW will provide methodological oversight and expertise and TC, AB, MK and MQ will provide feedback on the project protocol, assist with screening, data extraction and provide review of the manuscript.

## Eligibility criteria

All citations will be selected for inclusion according to the eligibility criteria presented below.

### Inclusion Criteria

STUDY DESIGN: All evidence synthesis (e.g., systematic reviews, rapid reviews, scoping reviews, and systematic review meta-analyses) will be included if they meet the minimum criteria suggested in the PRISMA-P definition of a systematic review (10, 13). That is, reviews explicitly stated methods to identify studies (I.e., search strategy methods and search dates), methods used for study selection (I.e., eligibility criteria and study selection process) and a narrative or quantitative synthesis of evidence.

PUBLICATION RANGE:

Evidence synthesis prior to the 2022 outbreak: Inception to April 30, 2022.

Evidence synthesis produced during the 2022 outbreak: 1 May 2022 and 31 December 2022.

POPULATION: Evidence synthesis evaluating human populations of any age.

INTERVENTION: Evidence synthesis evaluating any intervention/exposure or no intervention.

COMPARATORS: Evidence synthesis question may not have included studies with a comparator group.

OUTCOMES: Mpox evidence syntheses reporting one or more of the following outcomes: transmission efficiency, estimates of outbreak size, secondary attack rate, modes of transmission, viral kinetics, asymptomatic/pre-asymptomatic transmission, infectious period, incubation period, serial interval, serological data, clinical characteristics (symptomology and duration), severity risk factors, mortality, mortality risk factors, infection-induced immunity, pre- and post-exposure vaccination (e.g., vaccine effectiveness, break-through infections, safety), experimental studies on vaccine candidates, therapeutics, infection, prevention and control (IPC), diagnostic test accuracy, genomics and structural characterization (e.g., mutations, phylogenetic analyses, and protein-protein interactions), emergence and spread, public health measures (PHMs), adherence to PHMs, and knowledge, attitudes and behaviors (KAB).

LANGUAGE: English and French articles. French articles will be translated using Google translate.

### Exclusion criteria

Evidence syntheses not on mpox or human populations; published in languages other than English or French; primary literature; narrative reviews/non-evidence synthesis literature reviews, other non-primary literature (e.g., commentaries, letters to the editor, evidence synthesis protocols, etc.), overviews of reviews. Evidence syntheses that do not meet the minimum inclusion criteria will also be excluded.

## Information sources and search strategy

A database of all mpox literature was established in May 2022. A comprehensive search strategy that was developed and tested through an iterative process by an experienced information specialist in consultation with the review team and peer-reviewed by international colleagues. PubMed, Scopus, EuropePMC, SSRN and arXiv were searched twice weekly between 1 May 2022 to December 31, 2022 to identify preprint and published literature on mpox. The searches utilized keywords (e.g., monkeypox, mpox, simianpox, MPXV, variole du singe, and variole simienne). There were no language restrictions, but results were limited to the period of 14 April 2022 which was one month prior to the outbreak and 31 December 2022. The search algorithms were adapted to each respective database. Since May 2022, results of all primary and non-primary literature were maintained in DistillerSR (Evidence Partners, 2023) and a searchable excel database.

For this study, all citations previously screened as non-primary during the mpox literature surveillance will be rescreened for this project to classify the reviews as evidence syntheses or not (Appendix 1).

Historical synthesis research was not captured by the mpox literature surveillance for the 2022 outbreak. Thus, a secondary search from inception to April 30, 2022 will be conducted to identify all evidence synthesis prior to the 2022 outbreak. Additional keywords will be included to target evidence syntheses (e.g., scoping review, meta-analysis, systematic review and rapid review). The search strategy can be found in Appendix 2.

### Grey literature

A grey literature search will be conducted for both pre 2022 outbreak and 2022 outbreak evidence syntheses using targeted governmental websites including the World Health Organization, European Centre for Disease Prevention and Control, Public Health Agency of Canada, Centre of Disease Control and Prevention, and United Kingdom and the Health Security Agency. The grey literature search will be augmented by searching other electronic databases including OpenGrey and Google using keyword/phrase search that complements the bibliographic database search. The first 100 hits from each source will be examined from each search for relevance.

Search verification

Bibliographies of relevant evidence syntheses will be scanned for relevant citations omitted by the electronic and grey literature searches.

## Study selection

### Review management

Citations from the bibliographic databases and grey literature were collated in Endnote (Clarivate, Philadelphia, Pennsylvania, USA). De-duplication of articles were first conducted in Endnote and then deduplicated search results were uploaded to DistillerSR. DistillerSR will be used to manage relevance screening, study characterization, data extraction as well as methodological and reporting quality of citations levels of the project.

### Article screening

Screening of title and abstracts of potentially relevant citations will be conducted independently by two reviewers using the relevance screening form developed to reflect the inclusion and exclusion criteria. At title and abstract screening, a pilot test of 5% of citations will be conducted by all reviewers prior to screening citations to ensure consistency by allowing for modifications and clarifications to be made prior to commencing. The relevance screening form is included in Appendix 3.

Full-text screening will be conducted independently and in duplicate based on the eligibility criteria using a pre-determined form included in Appendix 3. At full text screening, 10% of the citations will be pre-tested and the forms will be adjusted until there is consensus with a good reviewer agreement (kappa ≥ 0.8) to ensure consistency by allowing for modifications and clarifications to be made prior to commencing. Disagreements will be resolved by consensus or by consulting with a senior reviewer. Reasons for exclusion at full text will be documented.

Articles that are not available electronically will be ordered via the Public Health Agency of Canada library and those that are unavailable will be reported.

## Article characterization

A data characterization form to extract key information from the studies is provided in Appendix 4. The form will be pilot-tested by all reviewers on a random sample of three articles (5%) and adjusted as needed. Data will be extracted from all studies selected for inclusion independently by two reviewers. Any disagreements will be resolved by consensus or third-party adjudication.

Data extraction elements:

- Number of authors
- Country of first author
- Publication date
- Language of publication
- Review definition and nomenclature
- Date of pre-print, submission and acceptance
- Purpose of study
- Eligibility criteria
- Search methods (e.g., search dates, databases, language restrictions, number of reviewers for study selection/extraction/quality assessment)
- Publication dates of included studies and grey literature
- Source(s) of funding
- Number of included reviews
- Study designs of included studies
- Study population characteristics
- Outcomes/Variables
- Use of Grading of Recommendations Assessment, Development and Evaluation (GRADE)
- Study limitations

## Quality and reporting

Methodological quality will be assessed using A Measurement Tool to Assess Systematic Reviews 2.0 (AMSTAR-2)(9). This is a critical appraisal tool for systematic reviews of randomized and/or non-randomized studies used determine how well the review is conducted. AMSTAR-2 consists of 16 domains including seven critical domains (I.e., domains 2, 4, 7, 9, 11, 13, and 15) and nine non-critical domains. A post hoc decision was made prior to analysis to consider publication bias (domain 15), a non-critical domain, since many included studies did not perform a MA and of those that did, there was a lack of power due to limited number of included studies to investigate publication bias. Domains 9 and 13 evaluating risk of bias (ROB) were ‘not applicable’ for syntheses that did not conduct ROB, similar to previously published studies (Baumeister et al., 2021; Kelly et al., 2016). Each item will be evaluated using ‘yes’, ‘partial yes’, or ‘no’. The overall quality will be evaluated using ‘critically low’, ‘low’, ‘moderate’ or ‘high’. There are no validated tools to assess AMSTAR for rapid and scoping reviews, thus AMSTAR-2 will be used as is for rapid reviews and will be adapted for scoping reviews (e.g., domain 9 and 13 evaluating ROB will be designated ‘not applicable’), similar to previously published studies(14, 15).

Reporting quality will be assessed using the PRISMA 2020 statement which consists of 27 items and developed to determine the strength of reporting of systematic reviews and meta-analysis(16). Response options include ‘yes’, ‘no’ and ‘not applicable’. Each “yes” will be designated 1 count for a maximum score of 27. Where available, extension of PRISMA will be used (e.g., PRISMA-Scr for scoping reviews (17)) and where extensions are not available, as in the case of rapid reviews PRISMA will be used to assess reporting quality. We will also assess rapid reviews, systematic reviews and scoping reviews using the PRISMA-Abstracts extension to evaluate quality of abstracts(16).

Quality assessment will be conducted independently by two reviewers. Disagreements will be resolved by consensus or third-party adjudication with a senior reviewer.

## Data synthesis

Data including study characteristics and quality assessment will be synthesized narratively and summarized using descriptive statistics (e.g., frequency, percentage, and proportions) as well as groupings of categories (e.g., number of authors and databases). Characteristics will be analysed as a whole and by type of evidence synthesis (e.g., SRs, MAs, ScRs, RRs). When necessary, a graphical display of the evidence will be provided in figures and tables. Syntheses published prior to May 2022 (historical) were contrasted with syntheses published May 2022 onwards (new). Some syntheses had incorrectly named study designs of their included primary studies. Thus, a post-hoc decision was made to re-classify these studies into appropriate study design categories for the analysis.

# Appendix 1. Search strategies for mpox studies published during the 2022 outbreak

The search algorithm applied during the 2022 mpox outbreak literature surveillance project was adapted to each database:

**Pubmed**

Monkeypox[All Fields] OR Monkeypox*[All Fields] OR "Monkey pox*"[All Fields] OR "Monkey orthopox*"[All Fields] OR Simianpox*[All Fields] OR "Simian pox*"[All Fields] OR "Simian orthopox*"[All Fields] OR MPXV[All Fields] OR Monkeypox[MeSH Terms] OR Monkeypox virus[MeSH Terms] OR "Variole du singe"[All Fields] OR "orthopoxvirose simienne"[All Fields] OR “Variole simienne”[All Fields] OR “mpox” [All Fields] AND ("2022/04/14"[PDAT] : "2023/12/31"[PDAT])

**Scopus**

TITLE-ABS-KEY (Monkeypox OR “Monkey pox” OR “Monkey orthopox” OR Simianpox OR “Simian pox” OR “Simian orthopox” OR MPXV OR “Monkeypox virus” OR “Variole du singe” OR “mpox” OR “orthopoxvirose simienne” OR “Variole simienne”) AND ORIG-LOAD-DATE > 20220414

**EuropePMC**

(Title:(Monkeypox) OR Title:("Monkey pox") OR Title:("Monkey orthopox") OR Title:(Simianpox) OR Title:("Simian pox") OR Title:("Simian orthopox") OR Title:(MPXV) OR Title:("Variole du singe") OR Title:("orthopoxvirose simienne") OR Title:("Variole simienne") OR Title:(“mpox”) OR Abstract:(Monkeypox) OR Abstract:("Monkey pox") OR Abstract:("Monkey orthopox") OR Abstract:(Simianpox) OR Abstract:("Simian pox") OR Abstract:("Simian orthopox") OR Abstract:(MPXV) OR Abstract:("Variole du singe") OR Abstract:("orthopoxvirose simienne") OR Abstract:("Variole simienne") OR Abstract:(“mpox”)) AND (SRC:PPR) AND (FIRST_PDATE:[2022-04-14 TO 2023-12-31])

**SSRN and ArXiv**

Search for the following keywords: Monkeypox, Simianpox, Variole du singe and Variole simienne, mpox as individual searches.

Appendix 2. Search strategies for mpox evidence syntheses published prior to the 2022 outbreak**Pubmed**

("meta-analysis"[All Fields] OR "systematic review"[All Fields] OR “scoping review”[All Fields] OR “rapid review”[All Fields]) OR (rapidreview* OR systematic* OR scoping* OR meta-analy*) AND (Monkeypox[All Fields] OR Monkeypox*[All Fields] OR "Monkey pox*"[All Fields] OR "Monkey orthopox*"[All Fields] OR Simianpox*[All Fields] OR "Simian pox*"[All Fields] OR "Simian orthopox*"[All Fields] OR MPXV[All Fields] OR Mpox[All Fields] OR Monkeypox[MeSH Terms] OR Monkeypox virus[MeSH Terms] OR "Variole du singe"[All Fields] OR "orthopoxvirose simienne"[All Fields] OR “Variole simienne”[All Fields] OR “mpox” [All Fields]) AND ("1000/01/01"[PDAT] : "2022/05/01"[PDAT]).

**Scopus**

TITLE-ABS-KEY ((“Meta-analysis” OR “systematic review” OR “scoping review” OR “rapid review”) AND (Monkeypox OR “Monkey pox” OR “Monkey orthopox” OR Simianpox OR “Simian pox” OR “Simian orthopox” OR MPXV OR “Monkeypox virus” OR “Variole du singe” OR “mpox” OR “orthopoxvirose simienne” OR “Variole simienne”)) AND ORIG-LOAD-DATE < 20220501

**EuropePMC**

((“Meta-analysis” OR “systematic review” OR “scoping review” OR “rapid review”) AND (Title:(Monkeypox) OR Title:("Monkey pox") OR Title:("Monkey orthopox") OR Title:(Simianpox) OR Title:("Simian pox") OR Title:("Simian orthopox") OR Title:(MPXV) OR Title:("Variole du singe") OR Title:("orthopoxvirose simienne") OR Title:("Variole simienne") OR Title:(“mpox”) OR Abstract:(Monkeypox) OR Abstract:("Monkey pox") OR Abstract:("Monkey orthopox") OR Abstract:(Simianpox) OR Abstract:("Simian pox") OR Abstract:("Simian orthopox") OR Abstract:(MPXV) OR Abstract:("Variole du singe") OR Abstract:("orthopoxvirose simienne") OR Abstract:("Variole simienne") OR Abstract:(“mpox”)) AND (SRC:PPR) AND (FIRST_PDATE:[1000-01-01 TO 2022-05-01]))

**SSRN and ArXiv**

Search for the following keywords: Monkeypox, Simianpox, Variole du singe and Variole simienne, mpox, systematic review, rapid review, scoping review as individual searches.

## Appendix 3. Relevance screening form

| **#** | **Question** | **Options** | **Definitions/Comments** |
| --- | --- | --- | --- |
| **Level 1 Screening** | | | |
| 1 | Is this citation an evidence synthesis on mpox? | - Yes - No (EXCLUDE) | *If you cannot tell whether the article is irrelevant, include and the full text can be evaluated at level 2.*  INCLUDE: All evidence synthesis on mpox as per the definition below or if they self-identified as an evidence synthesis (e.g., systematic review, scoping review, rapid review, systematic-review meta-analysis, etc.).  Evidence synthesis are defined as those that explicitly stated methods to identify studies (I.e., search strategy methods and search dates), methods used for study selection (I.e., eligibility criteria and study selection process) and a narrative or quantitative synthesis of evidence.  EXCLUDE**:** Evidence synthesis not on mpox or human populations. All primary literature; narrative reviews/non-evidence synthesis literature reviews, other non-primary literature (e.g., commentaries, letters to the editor, evidence synthesis protocols, etc.), overviews of reviews and reviews not on mpox. |
| **Level 2 Screening Form** | | | |
| 2 | Does this study describe an evidence synthesis? | - Yes- evidence synthesis - No – Narrative review - No - Umbrella review - No – Commentary with no evidence synthesis - No - Letter to the editor or opinion with no evidence synthesis - No-correspondence with no evidence synthesis - No - Evidence synthesis protocol - No-bibliomentric analysis - No – other non-primary sources (specify) | Based on reviewer assessment:  INCLUDE: All evidence synthesis evaluating primary research. Evidence synthesis is defined as : reviews explicitly stated methods to identify studies (I.e., search strategy methods and search dates), methods used to for study selection (I.e., eligibility criteria and study selection process) and a narrative or quantitative synthesis of evidence.  EXCLUDE**:** All primary literature; narrative reviews/non-evidence synthesis literature reviews, non-primary evidence syntheses, other non-primary literature (e.g., commentaries, bibliometric analysis, letters to the editor, evidence synthesis protocols, etc.) and overviews of reviews (i.e., umbrella reviews). |
|  | Hidden question (if no to Q2): Was the study mislabeled? | - Yes (___TXT___) - No | Select yes if the study was mislabelled as an evidence synthesis.  Select no is the authors labelled the study as a review only (e.g. the study is actually a narrative review and the authors called it a review) or labelled it correctly. |
| 3 | Does the evidence synthesis describe human populations? | - Yes - No (EXCLUDE) | INCLUDE: All syntheses that include human populations  EXCLUDE: All reviews that report on zoonotic/animal populations exclusively or are not directly on human health. |
| 4 | Is the study published in English or French? | - Yes - No (EXCLUDE) | INCLUDE: All English and French studies  EXCLUDE: All articles published in other languages. |
| 5 | Is the synthesis published during the 2022 mpox outbreak? | - Yes - No | Historical evidence refers to syntheses conducted prior to May 1, 2022 while those conducted May 1, 2022 onwards are during the 2022 outbreak.  Yes = 2022 outbreak evidence  No = historical evidence |

## Appendix 4. Data characterization form

| **#** | **Question** | **Options** | **Definitions/Comments** |
| --- | --- | --- | --- |
| **General Study Characteristics** | | | |
| 1 | How many authors does the review have? | *drop down menu of numbers* | Report the number of authors. Option to add number as they appear. |
| 2 | What type of report is this? | - Peer reviewed journal article - Letter to the editor - Commentary/ correspondence - Pre-print - Add as you go | Pre-print: the version of a scientific manuscript posted on a public server prior to formal peer review. |
| 3 | Fill in the following dates: | Pre-print date: ___TXT___  Submission date: ___TXT___  Accepted date: ___TXT___  Publication date: ___TXT___ | Include the full date in the following format: day, month, year (e.g., 12 June 2023 or 01 August 2022). In the case that the date is not available, month and year will be inputted.  State N/A if a date is not mentioned.  Publication date should be first published online date as it is the indexed date. |
| 4 | What language is this study published in? | - English - French | Select language. |
| 5 | What country is the first author from? | - Canada - US - UK - Australia - China - Add as needed - Not reported | Report affiliation of first author. |
| 6 | What are the source(s) of funding? | - Pharmaceutical company - Government - Academic and hospitals settings - Other (specify _TXT__) - None - Not reported | State if any source(s) of funding were reported in the review. Select *Not reported* if the authors do not mention any source of funding. |
| **Review Characteristics** | | | |
| 7 | What is the purpose/rationale for conducting the review? | ___TXT___ | State what the authors report is their rationale/objective for conducting the review.  Include any details on rationale regarding why a systematic review was done (I.e., in the context of what is or is not known). |
| 8 | What are the search dates within this review? | ___TXT___ | Include the full date in the following format: 12 June 2022 – 15 September 2022 or NR – 15 September 2022 if initial date range is not reported.  In the case that the date is not available, month and year will be inputted (e.g., May to September 2022).  If the dates are not provided, please indicate it as NR. |
| 9 | What number of databases were searched in the review? | - 1 - 2 - 3 - 4 - Add as needed | Select the number of databases searched through a systematic search only. Hand searched databases not included in this count. |
| 10 | What databases were searched in this review? | - PubMed - PubMed Central - Scopus - MEDLINE - Web of Science - Cochrane Library - Science Direct - Embase - EuropePMC - MedRxiv - BioRxiv - SSRN - Research square - Add as needed | Select as many databases searched through a systematic search only as are reported in the review. List hand searched databases under other. |
| 11 | Did study conduct a grey literature search? If so, indicate the search dates. | - Yes (specify___TXT___) - No – but rationale provided Not reported | Include the full date in the following format: 12 June 2022 – 15 September 2022.  In the case that the date is not available, month and year will be inputted (e.g., May to September 2022).  If the dates are not provided, please indicate it as NR. |
| 12 | Did the study search other sources of evidence?  If so, indicate which sources were searched. | - Yes (specify __TXT__) - No – but rationale provided - Not reported | If yes, specify which sources were searched (e.g., reference lists of included studies or relevant systematic reviews, contacting experts). |
| 13 | What is the inclusion criteria of the review? | ___TXT___ | State the inclusion criteria of the review |
| 14 | What is the exclusion criteria of the review? | ___TXT___ | State the exclusion criteria of the review |
| 15 | Did the review have any language restrictions in study selection? | - Yes - No - Not reported | Select if language restrictions were used. If no restrictions, select no. |
| 16 | How many studies did the review include in total? | - 1 - 2 - 3 - 4 - 5 - 6 - Add as needed | What was the total number of studies included? |
| 17 | State the number of studies that were published pre-outbreak (before May 1^st^, 2022) in the review | - 1 - 2 - 3 - 4 - 5 - 6 - 7 - Add as needed | Pre-outbreak refers to before May 1^st^, 2022. Reviewers will have to go through the individual 2022 studies to see when they were published. |
| 18 | State the number of studies that were published during the outbreak (after May 1^st^, 2022) in the review | - 1 - 2 - 3 - 4 - 5 - 6 - 7 - Add as needed | During the outbreak refers to after May 1^st^, 2022. |
| 19 | What is the publication date of earliest study included in the review? | ___TXT___ | Include the year in the following format: 2022). |
| 20 | What is the publication date of most recent study included in the review? | ___TXT___ | Include the year in the following format: (e.g., 2022). |
| 21 | What study designs were included in the review? | Presented in a table:   - Observational study - Case series - Case report - Retrospective Cohort - Prospective Cohort - Case control - Cross-sectional - Prevalence survey - Surveillance/longitudinal study - Cluster Investigation - Other OBS: ___TXT___ - Experimental study - Controlled trial - Challenge trial - Quasi-experiment - In-vitro studies - In-silico studies - Other EXP:___TXT___ - Predictive/Mathematical models - Qualitative study - Economic analysis - Diagnostic test accuracy - Phylogenetic analysis - Ecological study - Expert elicitation - Other: specify ____ - Not reported   For each outcome indicate the following:  What was the total number of citations with this study design?   - 1 - 2 - 3 - 4 - Add as needed   How many citations with this study design were published during the outbreak (May 1^st^, 2022 and beyond)?   - 1 - 2 - 3 - 4 - Add as needed | Select as many study designs as reported in the review. Select *Not reported* if the review does not indicate the study designs of the included studies  **Observational study:** assignment of subjects into a treated group versus a control group is natural (outside the control of the investigator).   - **Case series/report:** an in depth evaluation of one or more cases and their clinical history/background to build a picture of the natural history of a disease. Only cases of mpox described. - **Retrospective cohort:** longitudinal study in which investigators look back to assess individuals with a common exposure. - **Prospective Cohort:** - **Cohort study**: a study in which one or more groups of individuals with differing exposures to a suspected risk factor/predictor are observed and followed through time for occurrence of an outcome. - **Case-control study**: compares exposure to the risk factor/predictor in subjects who have an outcome ('cases') with those who don’t have the outcome, but are otherwise similar ('controls') and drawn from the same sampling frame. - **Cross-sectional:** one set of observations is collected at a single time point to examine prevalence of exposures, risk factors or disease. Ideally the sample is representative of the target population. - **Prevalence study:** a measurement of the outcome (disease) at a point in time on a representative sample of the target population. - **Surveillance/longitudinal study:** the on-going sampling from a defined representative sample of the target population to evaluate changes over time.   **Experimental study:** each subject is assigned to a treated group or a control group before the start of the treatment.   - **Challenge trial** is a controlled trial that includes exposure to the agent - **RCT:** an experimental study in which people are allocated to intervention/comparison groups using random methods - ***In vitro* study:** *in vitro* refers to performing a given procedure in a controlled environment outside of a living organism (e.g. in a test-tube, well, petri-dish etc.). This category will include studies using cell lines which as sometimes called *Ex vivo* studies instead of *In vitro*, but this is not consistent. - **In silico study:** performed via simulation on a computer - **Predictive/mathematical model:** a process that uses data mining and probability to forecast outcomes. - **Qualitative study:** aimed at understanding social phenomena, exploring issues, and answering questions of “why” and “how” (e.g. focus groups, interviews, surveys, self-reports, observations, document analysis). - **Economic analysis:** aimed at assessing cost and cost effectiveness of interventions. - **Diagnostic test accuracy:** summarizes evidence about test accuracy (e.g. those with a disease and those without). - **Phylogenetic analysis**: systematic study of reconstructing the past evolutionary history of extant species or taxa, based on present-day data, such as morphologies or molecular information (sequence data). - **Cluster investigation**: process of investigating a group or cluster of cases of a particular disease or health condition occurring in a defined population or geographic area. - **Ecological study:** the unit of observation is population or community - **Expert elicitation:** process of gathering and incorporating expert opinions, judgments, or knowledge on a particular topic or problem |
| 22 | Did the authors draw contrasts/comparisons between the 2022 outbreak evidence and historical evidence? | - Yes (___TXT___) - No - NA | If yes, indicate the outcomes and findings of what was compared/ contrasted as reported by authors. If not described, select no. If no new 2022 studies, select NA. |
| 23 | How many reviewers assessed study screening? | - 1 - 2 - Not reported - Other (specify __TXT__) | Select *not reported* if the authors did not state how many reviewers there were. Select O*ther for* modified methods for screening and extraction(e.g., two reviewers where one only spot checked) |
| 24 | How many reviewers assessed study extraction? | - 1 - 2 - Not reported - Other (specify __TXT__) | Select *not reported* if the authors did not state how many reviewers there were. Select O*ther for* modified methods for screening and extraction(e.g., two reviewers where one only spot checked) |
| 25 | How many reviewers did quality assessment? | - 1 - 2 - Not reported - N/A- No QA conducted - N/A – scoping review - Other (specify__TXT__) | Select *not reported* if the authors did not state how many reviewers there were. Select O*ther for* modified methods for screening and extraction(e.g., two reviewers where one only spot checked) |
| 26 | What type of review is it (as reported by the authors)? | - Systematic review (SR) - Systematic review –meta analysis (SR-MA) - Rapid review - Rapid review - meta analysis - Meta-analysis - Scoping Review - Living SR - Living SR-MA - Add as needed | Select a single option:  Use the "add" option if the researchers give a unique term to their synthesis research (e.g., rapid systematic review, systematic scoping review). |
| 27 | What type of review is it (as assessed by the reviewer)? | - Systematic review - Systematic review –meta analysis (SR-MA) - Rapid Review - rapid review - meta analysis - Meta-analysis - Scoping Review - Living Systematic Review - Living SR-MA | Based on the definitions provided, select what type of review that you think it is.  **Systematic Review** –  defined as studies that have a comprehensive search strategy (atleast 2 databases, search dates), methods for study selection (I.e., eligibility criteria and study selection process which includes independent and duplicate screening/extraction), a narrative or quantitative synthesis of evidence and quality assessment.  Note: Grey literature and reference list search is not mandatory  **Rapid Reviews** – as per Cochrane, it is a form of knowledge synthesis that accelerates the process of conducting a traditional systematic review through streamlining or omitting specific methods to produce evidence for stakeholders. For example, single reviewer for screening/extraction, not conducting quality assessment etc.    **Meta-Analysis** – as per the Cochrane library, meta-analysis is when results of the individual studies are combined to produce an overall statistic. This can be done by collecting data from more than one trial and combining them to generate an average result. This aims to provide a more precise estimate of the effects of an intervention and to reduce uncertainty.    **Scoping Review** – this type of review aims to map all the existing literature concerning volume, nature, and characteristics of the primary research (Arksey and O'Malley,2005). These reviews examine the extent, range, and nature of research activity in a topic area; determine the value and potential scope and cost of undertaking a full systematic review; summarize and disseminate research findings and identify research gaps in the existing literature.  **Living Reviews** - as per the Cochrane library, these types of reviews are either a form of rapid or systematic review which is continually updated, incorporating relevant new evidence as it becomes available and this process is monitored actively*.* |
| 28 | What is the study population includedin this review? | - General population - Gay, bisexual, men who have sex with men (gbMSM) - Children (specify __TXT__) - Pregnant women (specify __TXT__) - Hospitalized patients (in-patents) (specify __TXT__) - Elderly (specify __TXT__) - Immunocompromised (specify __TXT__) - Healthcare workers(specify __TXT__) - Other: ___TXT___ | Select as many populations as described in the review.  **General population:** the sample is representative of the general public (without any focus on specific demographic factors, behavioral factors, geographic region, etc.).  **Specific subgroup:** the sample is defined by the author. The study focuses on a specific subgroup within the general population. Report how authors describe this population in the textbox.  Specify how authors defined their population for all populations except general population and gbMSM. |
| 29 | What topic areas are covered in the review? | - Transmission - Clinical data - Therapeutics - Infection, prevention and control - Public Health Measures - Diagnostics - Genomics - Knowledge, awareness and attitudes - Other (___TXT___) | **Therapeutics**: any therapeutics for the treatment of mpox.(e.g., antivirals tecovirimat, brincidofovir, cidofovir).This includes clinical use, safety and efficacy of mpox antiviral drugs  **Transmission**: how the mpox virus is spread from one individual or host to another. This includes modes/settings of transmission, zoonotic transmission, a- and pre-symptomatic transmission, transmission efficiency, secondary attack rate, estimates of outbreak size, serial interval, reproduction numbers, incubation period, infectious period, viral kinetics and  serological data.  **Clinical Data**: This includes clinical characteristics (symptomology and duration), virulence, severity, severity risk factors, mortality, and mortality risk factors.  **Infection, Prevention and Control:** This includes IPC measures in healthcare settings, home/ community and  **Public health measures.** Studies that evaluate or describe a non-medical intervention to reduce the spread of disease. This can include isolation, quarantine, contact tracing, hand hygiene, masking, and travel restrictions. Many of these will be predictive models, but it is possible to have both experimental and observational evidence that contributes to this foci.  **Diagnostics**: This includes diagnostic tests and detection performance, including PCR, wastewater analysis, rapid tests, tools for sequencing, detection assays, and predictive model assisted detection.  **Genomics**: includes studies of mpox virus mutations, structural characterization, genetic makeup and phylogeny. |
| 30 | What outcomes are reported in this review? | - Transmission efficiency ___TXT___ - Estimate of outbreak size/duration ___TXT___ - Secondary attack rate ___TXT___ - Modes of transmission ___TXT___ - Viral kinetics ___TXT___ - Asymptomatic or presymptomatic transmission ___TXT___ - Infectious period ___TXT___ - Incubation period ___TXT___ - Serial interval ___TXT___ - Serological data ___TXT___ - Clinical characteristics ___TXT___ - Severity ___TXT___ - Severity risk factors ___TXT___ - Mortality ___TXT___ - Mortality risk factors ___TXT___ - Infection-induced Immunity ___TXT___ - Pre-exposure vaccination ___TXT___ - Post-exposure vaccination ___TXT___ - Therapeutics ___TXT___ - Diagnostic test accuracy ___TXT___ - Genomics and structural characterization ___TXT___ - Emergence and spread ___TXT___ - Infection, prevention and control ___TXT___ - Public health measures ___TXT___ - Knowledge, attitudes and behaviors ___TXT___ - Other: ___TXT__ | Select as many outcomes as reported in the review. Specify in the textbox if a meta-analysis was conducted for the outcome and provide details. For example, if a meta-analysis was conducted for clinical characteristics, write “meta-analysis conducted for fever, myalgia, headache”.  **Transmission efficiency:** Often predictive or mathematical models that estimate the basic reproduction number R0 or effective reproduction number Rt. The studies evaluate how a pathogen will spread in the population at a specific time / under specified conditions.  **Estimate of outbreak size/duration**: Studies that predict or forecast outbreak size, usually based on mathematical models. E.g., modelling studies that demonstrate cases that were imported could result in a 40-fold increase in cases.  **Secondary attack rate:** Research that describes the proportion or percentage of susceptible individuals who become infected after being exposed to an index case.  **Modes of Transmission**: Research that reports ways in which the virus spreads from an infected host to an uninfected host. This could include airborne transmission, fomite transmission, direct contact, animal to human contact, and studies that describe transmission risk in different settings (e.g., workplace, schools, household). Each mode of transmission could be separated into sub-categories including transmission setting, type or by country were needed.  **Viral kinetics:** Studies that describe virus kinetics which included virus load at different points during infection, measures of the presence or concentration of viable virus, analysis of different types of samples from the host, the load and survival of the virus in environmental sampling studies which may report on different  sample types (e.g., air, surfaces, hospitals, community settings, etc.). This foci can be separated by sub-categories when needed.  **Asymptomatic or presymptomatic transmission**: Citations that report pre-symptomatic (i.e., before symptoms were recognized, but the host is already infectious) or asymptomatic (i.e., no symptoms during infection, but is infectious and can transmit the pathogen) transmission    **Infectious period:** Studies that describe the duration during which a host is capable of directly or indirectly transmitting an infectious agent.  **Incubation period:** Incubation period: Studies that report the interval between exposure to a pathogen and the appearance of the first symptoms of a disease. Latent period (not often reported): Studies that report the interval between exposure to a pathogen and becoming infectious.  **Serial interval**: Studies that describe the time from illness onset in the primary case (infected individual) to illness onset in the secondary case.  **Serological data:** diagnostic methods that to identify antibodies in the blood of an mpox case that has recovered  **Post-exposure vaccination**: Articles describing impact of post-exposure vaccination (i.e., vaccination after individuals have been exposed to the virus). This can include studies that assess post-exposure vaccine effectiveness from infection, protection against severe disease or mortality, and safety.  **Pre-exposure vaccination:**  Studies describing impact of pre-exposure vaccination (i.e., vaccination prior to individuals being exposed to the pathogen). This can include studies that assess pre-exposure vaccine effectiveness against breakthrough infections, protection against severe disease (hospitalization) or mortality and safety. Depending on the topic consider creating subgroups for historical (e.g., childhood) vs. recent vaccination, different types of vaccines, changes in dosing etc.  Therapeutics: any therapeutics studied for the treatment of mpox.(e.g., antivirals tecovirimat, brincidofovir, cidofovir). This includes clinical use, safety and efficacy of mpox antiviral drugs  **Clinical characteristics**: Includes studies that describe symptomology, frequency of symptoms, and duration of illness. These characteristics can be separated out into sub-categories. These are often descriptive studies initially, followed by cohorts that may be more analytical.  **Severity**: Articles that report describe disease severity, measure the risk and frequency of severe disease and report on the duration of severe disease typically measured by hospitalizations, ICU, mechanical ventilation and the length of stay at each level of care.  **Severity risk factors:** This include studies describing preexisting attributes of the host (e.g., age, sex or socioeconomic status) or health related factors that are associated with developing severe disease (e.g., being immunocompromised, having comorbidities). This may include being part of a high-risk group (e.g., immunocompromised individuals, pregnant, etc.). The risk factors can be separated out by sub-category.  **Mortality:** Articles that report the disease progression that leads to death or reports the frequency of mortality due to the disease.  **Mortality risk factors**: This include studies describing host attributes or health related risk factors that are associated with a higher risk of mortality from the disease (e.g., comorbidities).  **Infection-induced Immunity**: Studies describing infection-induced immunologic protection against reinfection. This can include studies that report the risk of reinfection, those that report neutralizing antibodies after infection to study what constitutes a robust immune response and how long it lasts.  **Therapeutics**: Research that evaluates treatments for the disease in an infected host or preliminary experimental studies including *in vitro, in vivo,* or *in silico* studies that evaluated binding of compounds to viral protein complexes.  **Diagnostic test accuracy**: Articles that describe identification of the pathogen through one or more tests: PCR, sequencing, culture, antibody and/or antigen detection assays, rapid tests, and computer assisted diagnosis. Typically these studies will provide some measure of the tests ability to correctly classify individuals that are diseased or not diseased or classify samples as the pathogen is present or not present. Measures include sensitivity, specificity, positive or negative predictive value, ROC curves, agreement between tests etc. They can be separated by test category and whether it is detection (e.g., wastewater monitoring) or diagnostic (e.g., to diagnose an infection in a human or animal.  **Genomics and structural characterization:** Studies that report phylogenetic analyses and mutations. This can include studies that describe whole genome sequencing to identify large deletions and *in silico* studies that predict changes in the structure of the [PATHOGEN] due to the mutations and its implications. Also, research that describes protein-inhibitor interactions such as binding affinity between an inhibitor and a potential antiviral target or studies.  **Emergence and spread:** Articles that report estimates of susceptibility in the population, estimates of spread, growth rate, models that extrapolate wastewater surveillance data and ecological niche modelling. For example, forecasting models that predict trend in cases, models that investigate impact of importation of disease, and studies on first or initial detection of a pathogen (e.g. clinical diagnosis vs. wastewater).  **Infection, prevention and control**: Studies that reported the evaluation of any infection, prevention and control methods in a healthcare setting, home or community. Also, studies that reported preliminary or experimental data on how well an intervention (e.g. disinfectant) could work under controlled conditions.  This could include *in vitro* studies evaluating effectiveness of intervention on pathogen inactivation such as hand sanitizers, ultraviolet-C whole room disinfection and heat or *in silico* simulation studies.  **Public health measures**: Studies that evaluate or describe a non-medical intervention to reduce the spread of disease. This can include isolation, quarantine, contact tracing, hand hygiene, masking, and travel restrictions. Many of these will be predictive models, but it is possible to have both experimental and observational evidence that contributes to this foci.  **Knowledge, attitudes and behaviors:** Studies that evaluated the knowledge, attitudes, and behaviours of the population about the disease, its prevention (including vaccination) and managing the disease. This can include knowledge, intention and motivation to vaccinate, attitudes towards the disease and public health measures, behaviours to reduce risk. Included within this category are internet trend analyses (analysis of tweets and opinions and public interest on the disease via social media data or Google Trend analysis), but these could also be their own sub-category. |
| 31 and  32 | Did authors report reasons why a meta-analysis was not conducted? | - Yes   - - Insufficient data - No tools available - Heterogeneity - Other (___TXT___) - Not reported - N/A-scoping review - N/A- meta analysis conducted | Select yes if authors indicated a meta-analysis was conducted. |
| 33  and  34 | Did the study report any limitations of the evidence in the review or review process? | - Yes   - - Quality of studies (___TXT___)     - Limited number of studies (____TXT____)     - Limited number of participants (___TXT___)     - Strength of conclusions/evidence (___TXT___)     - Lack of recent data (___TXT__)     - Limitations of the review process (___TXT___)     - Other: ___TXT___ - No/not reported | Select “yes” if the authors stated any limitations or constraints when conducting the review and report them.  Infer and categorize based on what authors reported in the limitations. Specify what authors reported.  Select “no” if the authors do not mention any limitations. |
| 35 | Did the study report whether a methodologist was included as an author? | - Yes - No | A methodologist is a contributing author specializing in evidence-based medicine, epidemiology, or statistics.  Select “yes” if the authors report in the study that a methodologist was included/consulted (for example, epidemiologist or expert involvement) in the review.  Note: There is no need to look up the authors. |
| 36  and 37 | Did the review include a risk of bias (RoB) assessment? If yes, specify which tools were used (check all that apply) | - Yes   - - Newcastle-Ottawa Scale (___TXT___)     - ROBINS-I (___TXT___)     - Joanna-Briggs critical appraisal tool (___TXT__)     - Self-specified: (__TXT__)     - Add as you go - No/Not reported (___TXT___) - N/A- scoping review | For each tool, specify what authors reported (e.g., JBI was used for case series).  Select *self-specified* if the authors created their own criteria for RoB assessment.  For studies that reported conducting a risk of bias assessment but provided no tools/results/reference to a tool, please select *No/Not reported* and specify in the textbox.  If scoping review, select NA. |
| 38 | Hidden question Q36 (if yes): Were there modifications made to the ROB tool by the authors? | - Yes (specify ___TXT___) - No | Indicate what authors modified in the ROB tool where reported.  Select no if the authors reference the modified tool. |
| 39 | Hidden question Q36 (if no): Did the authors report why a risk of bias assessment was not conducted? | - Yes (specify ___TXT___) - No |  |
| 40 | Did the authors describe the overall quality of the included studies? | - Yes (specify ___TXT___) - No - N/A-scoping review | Specify what the authors reported was the overall quality of the included studies.  If scoping review, select NA. |
| 41 | Did the review include a GRADE assessment? | - Yes - No - N/A-scoping review | Select yes if the authors included GRADE assessment.  If scoping review, select NA. |
| 42 | Hidden question for Q41 (if yes): Were there modifications to GRADE assessment used? | - Yes (specify ___TXT___) - No | Indicate what authors modified from the GRADE assessment where reported. |
| 43 | Hidden question for Q41 (if no): Did the authors state why a grade assessment was not conducted? | - Yes (specify ___TXT___) - No | State the author’s reasons for not conducting a grade assessment. |
| 44 | What were the main results of the review (as stated by the authors)? | ___TXT___ | State the main results of the review |
| 45 | Did the authors identify if previously published systematic reviews were available on this topic? | - Yes (___TXT___)   No | If yes, specify what was stated and previous reference. |
| 46 | Hidden question for Q45 (if no): Did the authors report why they didn’t identify any previously published systematic reviews? | - Yes (___TXT___) - No |  |

**Preferred Reporting Items for Systematic reviews and Meta-Analyses**

| **Section and Topic** | **Item #** | **Checklist item** | **Location where item is reported** |
| --- | --- | --- | --- |
| **TITLE** | | |  |
| Title | 1 | Identify the report as a systematic review. |  |
| **ABSTRACT** | | |  |
| Abstract | 2 | See the PRISMA 2020 for Abstracts checklist.  *Adjusted: Evaluated using the 2015 PRISMA abstract definition:  Provide a structured summary including as applicable, background, objectives, data sources, study eligibility criteria, participants, interventions, study appraisal and synthesis methods, results, limitations, conclusions, implications and key findings. Systematic review registration number. |  |
| **INTRODUCTION** | | |  |
| Rationale | 3 | Describe the rationale for the review in the context of existing knowledge. |  |
| Objectives | 4 | Provide an explicit statement of the objective(s) or question(s) the review addresses. |  |
| **METHODS** | | |  |
| Eligibility criteria | 5 | Specify the inclusion and exclusion criteria for the review and how studies were grouped for the syntheses. |  |
| Information sources | 6 | Specify all databases, registers, websites, organizations, reference lists and other sources searched or consulted to identify studies. Specify the date when each source was last searched or consulted. |  |
| Search strategy | 7 | Present the full search strategies for all databases, registers and websites, including any filters and limits used. |  |
| Selection process | 8 | Specify the methods used to decide whether a study met the inclusion criteria of the review, including how many reviewers screened each record and each report retrieved, whether they worked independently, and if applicable, details of automation tools used in the process. |  |
| Data collection process | 9 | Specify the methods used to collect data from reports, including how many reviewers collected data from each report, whether they worked independently, any processes for obtaining or confirming data from study investigators, and if applicable, details of automation tools used in the process. |  |
| Data items | 10a | List and define all outcomes for which data were sought. Specify whether all results that were compatible with each outcome domain in each study were sought (e.g. for all measures, time points, analyses), and if not, the methods used to decide which results to collect. |  |
|  | 10b | List and define all other variables for which data were sought (e.g. participant and intervention characteristics, funding sources). Describe any assumptions made about any missing or unclear information. |  |
| Study risk of bias assessment | 11 | Specify the methods used to assess risk of bias in the included studies, including details of the tool(s) used, how many reviewers assessed each study and whether they worked independently, and if applicable, details of automation tools used in the process. |  |
| Effect measures | 12 | Specify for each outcome the effect measure(s) (e.g. risk ratio, mean difference) used in the synthesis or presentation of results. |  |
| Synthesis methods | 13a | Describe the processes used to decide which studies were eligible for each synthesis (e.g. tabulating the study intervention characteristics and comparing against the planned groups for each synthesis (item #5)). |  |
|  | 13b | Describe any methods required to prepare the data for presentation or synthesis, such as handling of missing summary statistics, or data conversions. |  |
|  | 13c | Describe any methods used to tabulate or visually display results of individual studies and syntheses. |  |
|  | 13d | Describe any methods used to synthesize results and provide a rationale for the choice(s). If meta-analysis was performed, describe the model(s), method(s) to identify the presence and extent of statistical heterogeneity, and software package(s) used. |  |
|  | 13e | Describe any methods used to explore possible causes of heterogeneity among study results (e.g. subgroup analysis, meta-regression). |  |
|  | 13f | Describe any sensitivity analyses conducted to assess robustness of the synthesized results. |  |
| Reporting bias assessment | 14 | Describe any methods used to assess risk of bias due to missing results in a synthesis (arising from reporting biases). |  |
| Certainty assessment | 15 | Describe any methods used to assess certainty (or confidence) in the body of evidence for an outcome. |  |
| **RESULTS** | | |  |
| Study selection | 16a | Describe the results of the search and selection process, from the number of records identified in the search to the number of studies included in the review, ideally using a flow diagram. |  |
|  | 16b | Cite studies that might appear to meet the inclusion criteria, but which were excluded, and explain why they were excluded. |  |
| Study characteristics | 17 | Cite each included study and present its characteristics. |  |
| Risk of bias in studies | 18 | Present assessments of risk of bias for each included study. |  |
| Results of individual studies | 19 | For all outcomes, present, for each study: (a) summary statistics for each group (where appropriate) and (b) an effect estimate and its precision (e.g. confidence/credible interval), ideally using structured tables or plots. |  |
| Results of syntheses | 20a | For each synthesis, briefly summarize the characteristics and risk of bias among contributing studies. |  |
|  | 20b | Present results of all statistical syntheses conducted. If meta-analysis was done, present for each the summary estimate and its precision (e.g. confidence/credible interval) and measures of statistical heterogeneity. If comparing groups, describe the direction of the effect. |  |
|  | 20c | Present results of all investigations of possible causes of heterogeneity among study results. |  |
|  | 20d | Present results of all sensitivity analyses conducted to assess the robustness of the synthesized results. |  |
| Reporting biases | 21 | Present assessments of risk of bias due to missing results (arising from reporting biases) for each synthesis assessed. |  |
| Certainty of evidence | 22 | Present assessments of certainty (or confidence) in the body of evidence for each outcome assessed. |  |
| **DISCUSSION** | | |  |
| Discussion | 23a | Provide a general interpretation of the results in the context of other evidence. |  |
|  | 23b | Discuss any limitations of the evidence included in the review. |  |
|  | 23c | Discuss any limitations of the review processes used. |  |
|  | 23d | Discuss implications of the results for practice, policy, and future research. |  |
| **OTHER INFORMATION** | | |  |
| Registration and protocol | 24a | Provide registration information for the review, including register name and registration number, or state that the review was not registered. |  |
|  | 24b | Indicate where the review protocol can be accessed, or state that a protocol was not prepared. |  |
|  | 24c | Describe and explain any amendments to information provided at registration or in the protocol. |  |
| Support | 25 | Describe sources of financial or non-financial support for the review, and the role of the funders or sponsors in the review. |  |
| Competing interests | 26 | Declare any competing interests of review authors. |  |
| Availability of data, code and other materials | 27 | Report which of the following are publicly available and where they can be found: template data collection forms; data extracted from included studies; data used for all analyses; analytic code; any other materials used in the review. |  |

**Preferred Reporting Items for Systematic reviews and Meta-Analyses 2020 for Abstracts**

| **Section and Topic** | **Item #** | **Checklist item** | **Reported (Yes/No)** |
| --- | --- | --- | --- |
| **TITLE** | | |  |
| Title | 1 | Identify the report as a systematic review. |  |
| **BACKGROUND** | | |  |
| Objectives | 2 | Provide an explicit statement of the main objective(s) or question(s) the review addresses. |  |
| **METHODS** | | |  |
| Eligibility criteria | 3 | Specify the inclusion and exclusion criteria for the review. |  |
| Information sources | 4 | Specify the information sources (e.g. databases, registers) used to identify studies and the date when each was last searched. |  |
| Risk of bias | 5 | Specify the methods used to assess risk of bias in the included studies. |  |
| Synthesis of results | 6 | Specify the methods used to present and synthesize results. |  |
| **RESULTS** | | |  |
| Included studies | 7 | Give the total number of included studies and participants and summarize relevant characteristics of studies. |  |
| Synthesis of results | 8 | Present results for main outcomes, preferably indicating the number of included studies and participants for each. If meta-analysis was done, report the summary estimate and confidence/credible interval. If comparing groups, indicate the direction of the effect (i.e. which group is favoured). |  |
| **DISCUSSION** | | |  |
| Limitations of evidence | 9 | Provide a brief summary of the limitations of the evidence included in the review (e.g. study risk of bias, inconsistency and imprecision). |  |
| Interpretation | 10 | Provide a general interpretation of the results and important implications. |  |
| **OTHER** | | |  |
| Funding | 11 | Specify the primary source of funding for the review. |  |
| Registration | 12 | Provide the register name and registration number. |  |

**Preferred Reporting Items for Systematic reviews and Meta-Analyses 2020 for Scoping Review Abstracts**

| **Section and Topic** | **Item #** | **Checklist item** | **Reported (Yes/No)** |
| --- | --- | --- | --- |
| **TITLE** | | |  |
| Title | 1 | Identify the report as a scoping review. |  |
| **BACKGROUND** | | |  |
| Objectives | 2 | Provide an explicit statement of the main objective(s) or question(s) the review addresses. |  |
| **METHODS** | | |  |
| Eligibility criteria | 3 | Specify the inclusion and exclusion criteria for the review. |  |
| Information sources | 4 | Specify the information sources (e.g. databases, registers) used to identify studies and the date when each was last searched. |  |
| Critical Appraisal ** | 5 | Specify the methods for conducting a critical appraisal of the included sources of evidence. ** |  |
| Synthesis of results | 6 | Specify the methods used to present and synthesize results. |  |
| **RESULTS** | | |  |
| Included studies | 7 | Give the total number of included studies and participants and summarize relevant characteristics of studies. |  |
| Synthesis of results | 8 | Summarize and present results as they relate to the review questions and objectives.** |  |
| **DISCUSSION** | | |  |
| Limitations** | 9 | Provide a brief summary of the limitations of the scoping review process. ** |  |
| Interpretation | 10 | Provide a general interpretation of the results and important implications. |  |
| **OTHER** | | |  |
| Funding | 11 | Specify the primary source of funding for the review. |  |
| Registration | 12 | Provide the register name and registration number. |  |

Note: Critical appraisal refers to the process of systematically examining research evidence to assess its validity, results, and relevance before using it to inform a decision. This term is used for items 12 and 19 instead of "risk of bias" (which is more applicable to systematic reviews of interventions) to include and acknowledge the various sources of evidence that may be used in a scoping review (e.g., quantitative and/or qualitative research, expert opinion, and policy document)

**adjusted for scoping reviews

**Preferred Reporting Items for Systematic reviews and Meta-Analyses extension for Scoping Reviews (PRISMA-ScR) Checklist**

| **SECTION** | **ITEM** | **PRISMA-ScR CHECKLIST ITEM** | **REPORTED ON PAGE #** |
| --- | --- | --- | --- |
| **TITLE** | | | |
| Title | 1 | Identify the report as a scoping review. | Click here to enter text. |
| **ABSTRACT** | | | |
| Structured summary | 2 | Provide a structured summary that includes (as applicable): background, objectives, eligibility criteria, sources of evidence, charting methods, results, and conclusions that relate to the review questions and objectives. | Click here to enter text. |
| **INTRODUCTION** | | | |
| Rationale | 3 | Describe the rationale for the review in the context of what is already known. Explain why the review questions/objectives lend themselves to a scoping review approach. | Click here to enter text. |
| Objectives | 4 | Provide an explicit statement of the questions and objectives being addressed with reference to their key elements (e.g., population or participants, concepts, and context) or other relevant key elements used to conceptualize the review questions and/or objectives. | Click here to enter text. |
| **METHODS** | | | |
| Protocol and registration | 5 | Indicate whether a review protocol exists; state if and where it can be accessed (e.g., a Web address); and if available, provide registration information, including the registration number. | Click here to enter text. |
| Eligibility criteria | 6 | Specify characteristics of the sources of evidence used as eligibility criteria (e.g., years considered, language, and publication status), and provide a rationale. | Click here to enter text. |
| Information sources* | 7 | Describe all information sources in the search (e.g., databases with dates of coverage and contact with authors to identify additional sources), as well as the date the most recent search was executed. | Click here to enter text. |
| Search | 8 | Present the full electronic search strategy for at least 1 database, including any limits used, such that it could be repeated. | Click here to enter text. |
| Selection of sources of evidence† | 9 | State the process for selecting sources of evidence (i.e., screening and eligibility) included in the scoping review. | Click here to enter text. |
| Data charting process‡ | 10 | Describe the methods of charting data from the included sources of evidence (e.g., calibrated forms or forms that have been tested by the team before their use, and whether data charting was done independently or in duplicate) and any processes for obtaining and confirming data from investigators. | Click here to enter text. |
| Data items | 11 | List and define all variables for which data were sought and any assumptions and simplifications made. | Click here to enter text. |
| Critical appraisal of individual sources of evidence§ | 12 | If done, provide a rationale for conducting a critical appraisal of included sources of evidence; describe the methods used and how this information was used in any data synthesis (if appropriate). | Click here to enter text. |
| Synthesis of results | 13 | Describe the methods of handling and summarizing the data that were charted. | Click here to enter text. |
| **RESULTS** | | | |
| Selection of sources of evidence | 14 | Give numbers of sources of evidence screened, assessed for eligibility, and included in the review, with reasons for exclusions at each stage, ideally using a flow diagram. | Click here to enter text. |
| Characteristics of sources of evidence | 15 | For each source of evidence, present characteristics for which data were charted and provide the citations. | Click here to enter text. |
| Critical appraisal within sources of evidence | 16 | If done, present data on critical appraisal of included sources of evidence (see item 12). | Click here to enter text. |
| Results of individual sources of evidence | 17 | For each included source of evidence, present the relevant data that were charted that relate to the review questions and objectives. | Click here to enter text. |
| Synthesis of results | 18 | Summarize and/or present the charting results as they relate to the review questions and objectives. | Click here to enter text. |
| **DISCUSSION** | | | |
| Summary of evidence | 19 | Summarize the main results (including an overview of concepts, themes, and types of evidence available), link to the review questions and objectives, and consider the relevance to key groups. | Click here to enter text. |
| Limitations | 20 | Discuss the limitations of the scoping review process. | Click here to enter text. |
| Conclusions | 21 | Provide a general interpretation of the results with respect to the review questions and objectives, as well as potential implications and/or next steps. | Click here to enter text. |
| **FUNDING** | | | |
| Funding | 22 | Describe sources of funding for the included sources of evidence, as well as sources of funding for the scoping review. Describe the role of the funders of the scoping review. | Click here to enter text. |

JBI = Joanna Briggs Institute; PRISMA-ScR = Preferred Reporting Items for Systematic reviews and Meta-Analyses extension for Scoping Reviews.

* Where *sources of evidence* (see second footnote) are compiled from, such as bibliographic databases, social media platforms, and Web sites.

† A more inclusive/heterogeneous term used to account for the different types of evidence or data sources (e.g., quantitative and/or qualitative research, expert opinion, and policy documents) that may be eligible in a scoping review as opposed to only studies. This is not to be confused with *information sources* (see first footnote).

‡ The frameworks by Arksey and O’Malley (6) and Levac and colleagues (7) and the JBI guidance (4, 5) refer to the process of data extraction in a scoping review as data charting*.*

§ The process of systematically examining research evidence to assess its validity, results, and relevance before using it to inform a decision. This term is used for items 12 and 19 instead of "risk of bias" (which is more applicable to systematic reviews of interventions) to include and acknowledge the various sources of evidence that may be used in a scoping review (e.g., quantitative and/or qualitative research, expert opinion, and policy document).

*From:* Tricco AC, Lillie E, Zarin W, O'Brien KK, Colquhoun H, Levac D, et al. PRISMA Extension for Scoping Reviews (PRISMAScR): Checklist and Explanation. Ann Intern Med. 2018;169:467–473. [doi: 10.7326/M18-0850](http://annals.org/aim/fullarticle/2700389/prisma-extension-scoping-reviews-prisma-scr-checklist-explanation).

AMSTAR

AMSTAR-2 consists of 16 domains including seven critical domains (I.e., domains 2, 4, 7, 9, 11, 13, and 15)

| 1 | Did the research questions and inclusion criteria for the review include the components of PICO/PECO? | - Population - Intervention (NA for certain review questions) - Exposure (NA for certain review questions) - Comparator Group (NA for certain review questions) - Outcome - Timeframe for follow-up (Optional)   Response   - Yes - No | Select the appropriate items based on the review question and downgrade only if necessary items are not reported.  Examples:  Studies that look at clinical data only:  YES- population and outcome and/or exposure reported. NO - If only population or only outcome reported  Studies that evaluate interventions::  YES – Population, intervention, comparator, outcome described  No- If any one of the above is not reported |
| --- | --- | --- | --- |
| 2 | *Did the report of the review contain an explicit statement that the review methods were established prior to the conduct of the review and did the report justify any significant deviations from the protocol?* | *For Partial Yes: The authors state that they had a written protocol or guide that included ALL of the following:*   - Review Questions - A search strategy - Inclusion/exclusion criteria - A risk of bias assessment (NA for scoping reviews).   For Yes: *As for partial yes, plus the protocol should be registered and should also have specified:*   - a meta-analysis/synthesis plan, if appropriate, and - A plan for investigating causes of heterogeneity - Justification for any deviations from the protocol   Response:   - Yes - Partial yes - No | Response for rapid reviews and systematic reviews:  For PARTIAL YES: all criteria are selected  For YES: all criteria plus partial criteria should be selected.  Exceptions for scoping reviews:  PARTIAL YES- review question, search strategy, inclusion/exclusion criteria needed  NO- if any one of the above is missing  FOR YES – all criteria have to be met |
| 3 | Did the review authors explain their selection of the study designs for inclusion in the review? | Does the review satisfy ONE of the following:   - Explanation for including only RCTs - OR Explanation for including only NRS - OR Explanation for including both RCTs and NRS   Response   - Yes - No | NRS: Non-randomised studies  For YES: the review satisfies at least 1 criteria |
| 4 | Did the review authors use a comprehensive literature search strategy? | *For Partial Yes: The authors state that they had a written protocol or guide that included ALL of the following:*   - searched at least 2 databases (relevant to research question) - Provided key word and/or search strategy - Justified publication restrictions (e.g. language)   *For Yes: As for partial yes, plus the protocol should be registered and should also have specified:*   - searched the reference lists/bibliographies of included studies - Searched trial/study registries - Included/consulted content experts in the field - Where relevant, searched for grey literature - Conducted search within 24 months of completion of review   Response   - Yes - Partial Yes - No | For PARTIAL YES, all of the items should be selected.  For YES, all of the items should be selected plus all the partial items should be selected. |
| 5 | Did the review authors perform study selection in duplicate? | Choose either ONE of the following:   - at least two reviewers independently agreed on selection of eligible studies and achieved consensus on which studies to include - OR two reviewers selected a sample of eligible studies and achieved good agreement (at least 80 percent), with the remainder selected by one reviewer   Response   - Yes - No | For YES, review should satisfy either one of the criteria. |
| 6 | Did the review authors perform data extraction in duplicate? | Choose either ONE of the following:   - at least two reviewers achieved consensus on which data to extract from included studies - OR two reviewers extracted data from a sample of eligible studies and achieved good agreement (at least 80 percent), with the remainder extracted by one reviewer   Response   - Yes - No | For YES, review should satisfy either one of the criteria. |
| 7 | Did the review authors provide a list of excluded studies and justify the exclusions? | *For Partial Yes:*   - provided a list of all potentially relevant studies that were read in full text form but excluded from the review   *For Yes, must also have:*   - justified the exclusion from the review of each potentially relevant study   Response   - Yes - Partial yes - No | For PARTIAL YES, review should satisfy all criteria.  For YES, review should satisfy all criteria as well as all partial criteria. |
| 8 | Did the review authors describe the included studies in adequate detail? | *For Partial Yes (ALL of the following):*   - Described populations - Described interventions (NA for certain review questions) - Described exposures (NA for certain review questions) - Described comparators (NA for certain review questions) - Described outcomes - Described research designs   *For Yes, should also have ALL the following:*   - described population in detail - described intervention and comparator in detail (including doses where relevant) (NA for certain review questions) - described exposure (NA for certain review questions) - described study's setting - timeframe for follow-up (NA for certain review questions)   Response:   - Yes - Partial Yes - No | **note: for research design, select only if reported somewhere in the study. May not necessarily be in the table of characteristics. Study settings should include details such as community or hospital settings not just country to designate a yes.*  Select the appropriate items based on the review question and downgrade only if necessary items are not reported.  Examples:  Studies that look at clinical data only:  For PARTIAL YES - population, outcome, research designs are ALL reported.  **note: Interventions/exposure and/or comparators may be reported for some review questions while other questions will not be reported. Score as NO, if these criteria pertain to the research question and are not reported and Score as PARTIAL YES if they do not pertain to the research question and are not reported.*  For YES, population described in detail and study setting.  **note: Interventions/exposure and/or comparators may be reported for some review questions while other questions will not be reported. Score as NO, if these criteria pertain to the research question and are not reported and Score as YES if they do not pertain to the research question and reported.*  Studies that evaluate interventions:  For PARTIAL YES – Population, intervention and/or exposure comparator, outcome, research designs all described  For NO, if any one of the above is not reported  For YES, review should satisfy population described in detail and described study settings).  **note: Interventions/exposure and/or comparators may be reported for some questions while other questions will not be reported. Score as NO, if these criteria pertain to the research question and are not reported and Score as YES if they do not pertain to the research question.* |
| 9 | RCTs- Did the review authors use a satisfactory technique for assessing the risk of bias (RoB) in individual studies that were included in the review? | *For Partial Yes, must have assessed RoB from:*   - unconcealed allocation, and - lack of blinding of patients and assessors when assessing outcomes (unnecessary for objective outcomes such as all-cause mortality)   *For Yes, must also have assessed RoB from:*   - allocation sequence that was not truly random, and - selection of the reported result from among multiple measurements or analyses of a specified outcome   Response   - Yes - Partial yes - No - Includes only NRS - NA - scoping review | Select NA for scoping reviews or if studies only include NRS, select NRS. |
| 9 | **NRS** - Did the review authors use a satisfactory technique for assessing the risk of bias (RoB) in individual studies that were included in the review? | *For Partial Yes, must have assessed RoB from:*   - *Confounding* - *Selection bias*   *For Yes, must also have assessed RoB from:*   - methods used to ascertain exposures and outcomes, and - selection of the reported result from among multiple measurements or analyses of a specified outcome (NA for certain review questions)   Response   - Yes - Partial yes - No - Includes only RCTs - NA – scoping review | For PARTIAL YES, must satisfy all criteria.  For NO, satisfies only one or none of the criteria.  For YES, review must satisfy both criteria except for NRS where review questions do not require multiple measurements, this item is not needed.  **note if at least one of the tools used assessed confounding or selection, they should be selected.* |
| 10 | Did the review authors report on the sources of funding for the studies included in the review? | For Yes, Must have reported on the sources of funding for individual studies included in the review. Note: Reporting that the reviewers looked for this information but it was not reported by study authors also qualifies.  Response:   - Yes - No |  |
| 11 | ***RCTs -*** If meta-analysis was performed did the review authors use appropriate methods for statistical combination of results? | *For Yes, must also have assessed RoB from:*   - The authors justified combining the data in a meta-analysis - AND they used an appropriate weighted technique to combine study results and adjusted for heterogeneity if present - AND investigated the causes of any heterogeneity   Response   - Yes - No - No meta-analysis conducted | If no RCTs included, skip question. |
| 11 | **NRS*-*** If meta-analysis was performed did the review authors use appropriate methods for statistical combination of results? | *For Yes, must also have assessed RoB from:*   - The authors justified combining the data in a meta-analysis - AND they used an appropriate weighted technique to combine study results, adjusting for heterogeneity if present - AND they statistically combined effect estimates from NRS that were adjusted for confounding, rather than combining raw data, or justified combining raw data when adjusted effect estimates were not available - AND they reported separate summary estimates for RCTs and NRS separately when both were included in the review (N/A if only NRS included)   Response   - Yes - No - No meta-analysis conducted | *note if no RCTs were included “AND they reported separate summary estimates for RCTs and NRS separately when both were included in the review” is NA. Do not downgrade. |
| 12 | If meta-analysis was performed, did the review authors assess the potential impact of RoB in individual studies on the results of the meta-analysis or other evidence synthesis? | *For Yes;*   - included only low risk of bias RCTs - OR, if the pooled estimate was based on RCTs and/or NRS at variable RoB, the authors performed analyses to investigate possible impact of RoB on summary estimates of effect   Score   - Yes - No - No meta-analysis conducted | For YES, review must satisfy either of the criteria listed. |
| 13 | Did the review authors account for RoB in individual studies when interpreting/discussing the results of the review? | For Yes:   - included only low risk of bias RCTs - OR, if RCTs with moderate or high RoB, or NRS were included the review provided a discussion of the likely impact of RoB on the results   Response:   - Yes - No - NA scoping review | For YES, review must satisfy either of the criteria listed. |
| 14 | Did the review authors provide a satisfactory explanation for, and discussion of, any heterogeneity observed in the results of the review? | *For Yes:*   - There was no significant heterogeneity in the results - OR if heterogeneity was present the authors performed an investigation of sources of any heterogeneity in the results and discussed the impact of this on the results of the review   Response   - Yes - No | For YES, review must satisfy either of the criteria listed.  Even for scoping reviews authors should discuss variability between some outcomes, even if it is not the quantitative measurements within studies. |
| 15 | If they performed quantitative synthesis did the review authors carry out an adequate investigation of publication bias (small study bias) and discuss its likely impact on the results of the review? | *For Yes:*   - performed graphical or statistical tests for publication bias and discussed the likelihood and magnitude of impact of publication bias   Response   - Yes - No - No meta-analysis conducted | For YES, review must satisfy the criteria listed. |
| 16 | D id the review authors report any potential sources of conflict of interest, including any funding they received for conducting the review? | *For Yes:*   - The authors reported no competing interests OR - The authors described their funding sources and how they managed potential conflicts of interest   Response   - Yes - No | For YES, review must satisfy either of the criteria listed. |
| 17 | Overall confidence in the results of the review | Response   - High - Moderate - Low - Critically low | From AMSTAR 2:  High:  No or one non-critical weakness the systematic/rapid/scoping review provides an accurate and comprehensive summary of the results of the available studies that address the question of interests  Moderate:  More than one non-critical weakness*: the systematic review has more than one weakness but no critical flaws. It may provide an accurate summary of the results of the available studies that were included in the review.  Low  One critical flaw with or without non-critical weaknesses: the review has a critical flaw and may not provide an accurate and comprehensive summary of the available studies that address the question of interest.  Critically low:  More than one critical flaw with or without non-critical weaknesses: the review has more than one critical flaw and should not be relied on to provide an accurate and comprehensive summary of the available studies.  *Multiple non-critical weaknesses may diminish confidence in the review and it may be appropriate to move the overall appraisal down from moderate to low confidence. |

## References:

1. World Health Organization. 2022-23 Mpox (Monkeypox) Outbreak: Global Trends 2023 [Available from: <https://worldhealthorg.shinyapps.io/mpx_global/#1_Overview>.

2. World Health Organization. Monkeypox 2023 [Available from: <https://www.who.int/health-topics/monkeypox#tab=tab_1>.

3. Foster SO, Brink EW, Hutchins DL, Pifer JM, Lourie B, Moser CR, et al. Human monkeypox. Bull World Health Organ. 1972;46(5):569-76.

4. Nalca A, Rimoin AW, Bavari S, Whitehouse CA. Reemergence of monkeypox: prevalence, diagnostics, and countermeasures. Clin Infect Dis. 2005;41(12):1765-71.

5. McCollum AM, Damon IK. Human monkeypox. Clin Infect Dis. 2014;58(2):260-7.

6. Beer EM, Rao VB. A systematic review of the epidemiology of human monkeypox outbreaks and implications for outbreak strategy. PLoS Negl Trop Dis. 2019;13(10):e0007791.

7. Bunge EM, Hoet B, Chen L, Lienert F, Weidenthaler H, Baer LR, et al. The changing epidemiology of human monkeypox-A potential threat? A systematic review. PLoS Negl Trop Dis. 2022;16(2):e0010141.

8. McDonald S, Turner SL, Nguyen PY, Page MJ, Turner T. Are COVID-19 systematic reviews up to date and can we tell? A cross-sectional study. Syst Rev. 2023;12(1):85.

9. Shea BJ, Reeves BC, Wells G, Thuku M, Hamel C, Moran J, et al. AMSTAR 2: a critical appraisal tool for systematic reviews that include randomised or non-randomised studies of healthcare interventions, or both. BMJ. 2017;358:j4008.

10. Moher D, Shamseer L, Clarke M, Ghersi D, Liberati A, Petticrew M, et al. Preferred reporting items for systematic review and meta-analysis protocols (PRISMA-P) 2015 statement. Syst Rev. 2015;4(1):1.

11. Thomson H. Improving utility of evidence synthesis for healthy public policy: the three Rs (relevance, rigor, and readability [and resources]). Am J Public Health. 2013;103(8):e17-23.

12. Tricco AC, Khalil H, Holly C, Feyissa G, Godfrey C, Evans C, et al. Rapid reviews and the methodological rigor of evidence synthesis: a JBI position statement. JBI Evid Synth. 2022;20(4):944-9.

13. Shamseer L, Moher D, Clarke M, Ghersi D, Liberati A, Petticrew M, et al. Preferred reporting items for systematic review and meta-analysis protocols (PRISMA-P) 2015: elaboration and explanation. BMJ. 2015;350:g7647.

14. Baumeister A, Corrin T, Abid H, Young KM, Ayache D, Waddell L. The quality of systematic reviews and other synthesis in the time of COVID-19. Epidemiol Infect. 2021;149:e182.

15. Kelly SE, Moher D, Clifford TJ. Quality of conduct and reporting in rapid reviews: an exploration of compliance with PRISMA and AMSTAR guidelines. Syst Rev. 2016;5:79.

16. Page MJ, McKenzie JE, Bossuyt PM, Boutron I, Hoffmann TC, Mulrow CD, et al. The PRISMA 2020 statement: an updated guideline for reporting systematic reviews. Rev Esp Cardiol (Engl Ed). 2021;74(9):790-9.

17. Tricco AC, Lillie E, Zarin W, O'Brien KK, Colquhoun H, Levac D, et al. PRISMA Extension for Scoping Reviews (PRISMA-ScR): Checklist and Explanation. Ann Intern Med. 2018;169(7):467-73.
