# Supplementary material for: Methodological and reporting quality of systematic and rapid reviews on human mpox and their utility during a public health emergency
Source: Cochrane Evid Synth Methods. 2024 Nov 15;2(11):e70005. doi: 10.1002/cesm.70005 (PMC11795912; doi:10.1002/cesm.70005)
Supplement: Supplementary file 3 — Supporting information. [file CESM-2-e70005-s006.docx]

**Supplementary 3: Total number of syntheses per topic area reported**
