# Supplementary material for: Methodological and reporting quality of systematic and rapid reviews on human mpox and their utility during a public health emergency
Source: Cochrane Evid Synth Methods. 2024 Nov 15;2(11):e70005. doi: 10.1002/cesm.70005 (PMC11795912; doi:10.1002/cesm.70005)
Supplement: Supplementary file 4 — Supporting information. [file CESM-2-e70005-s003.docx]

**Supplementary 4: Study designs of included studies across evidence syntheses**

| Study design | Characteristic | Systematic review | | Meta-analysis (N=1) | Scoping Review (N=4) | Rapid Review (N=2) | Other*  (N=5) | Total**  (N=21) |
| --- | --- | --- | --- | --- | --- | --- | --- | --- |
|  |  | Historical  (N=) | New  (N=12) |  |  |  |  |  |
| Case report | No. Syntheses that include study design  Median No. studies included per design (range) | 1  Total  9 (9-9) | 10  Total  4 (1-34)  2022 outbreak  3 (0-31) | 0 | 2  Total  2 (1-3)  2022 outbreak  1.5 (0-2) | 1  Total  18 (18-18)  2022 outbreak  NR | 2  Total  11 (6-16)  2022 outbreak  7 (3-11) | 16 (71%)  Total  5 (1-34)  2022 outbreak  2 (0-31) |
| Case series | No. Syntheses that include study design  Median No. studies included per design (range) | 0 | 11  Total  4 (1-10)  2022 outbreak  1.5 (1-10) | 1  Total  3 (3-3)  2022 outbreak  3 (3-3) | 2  Total  3 (1-5)  2022 outbreak  3 (1-5) | 1  Total  44 (44-44)  2022 outbreak  NR | 3  Total  2 (2-6)  2022 outbreak  2 (1-2) | 18 (86%)  Total  4 (1-44)  2022 outbreak  2 (1-10) |
| Cross-sectional | No. Syntheses that include study design  Median No. studies included per design (range) | 1  Total  2 (2-2) | 6  Total  1.5 (1-11)  2022 outbreak  1 (0-10) | 0 | 1  Total  15 (15-5)  2022 outbreak  15 (15-15) | 0 | 1  Total  1 (1-1)  2022 outbreak  0 | 8 (38%)  Total  2 (1-15)  2022 outbreak  1 (0-15) |
| Retrospective Cohort study | No. Syntheses that include study design  Median No. studies included per design (range) | 0 | 4  Total  1 (1-2)  2022 outbreak  1 (1-2) | 1  Total  1 (1-1)  2022 outbreak  1 (1-1) | 1  Total  1 (1-1)  2022 outbreak  1 (1-1) | 0 | 3  Total  4 (2-4)  New  1 (1-2) | 9 (35%)  Total  1 (1-4)  2022 outbreak  1 (1-2) |
| Prospective cohort study | No. Syntheses that include study design  Median No. studies included per design (range) | 0 | 4  Total  1.5 (1-2)  2022 outbreak  1.5 (1-2) | 1  Total  3 (3-3)  2022 outbreak  3 (3-3) | 1  Total  2 (2-2)  2022 outbreak  2 (2-2) | 0 | 2  Total  1 (1-1)  New  1 (1-1) | 8 (38%)  Total  1.5 (1-3)  2022 outbreak  1.5 (1-3) |
| Case-control | No. Syntheses that include study design  Median No. studies included per design (range) | 1  Total  2 (2-2) | 0 | 0 | 0 | 0 | 0 | 1 (5%)  Total  2 (2-2) |
| Predictive/  Mathematical model | No. Syntheses that include study design  Median No. studies included per design (range) | 1  Total  1 (1-1) | 0 | 0 | 0 | 0 | 0 | 1 (5%)    Total  1 (1-1) |
| Surveillance/longitudinal study | No. Syntheses that include study design  Median No. studies included per design (range) | 1  Total  43 (43-43) | 5  Total  3 (1-6)  2022 outbreak  3 (0-6) | 0 | 0 | 0 | 2  Total  3 (1-5)  2022 outbreak  2.5 (1-4) | 8 (38%)  Total  3.5 (1-43)  2022 outbreak  3 (0-6) |
| Clinical trial | No. Syntheses that include study design  Median No. studies included per design (range) | 0 | 0 | 0 | 1  Total  3 (3-3)  2022 outbreak  2 (2-2) | 0 | 0 | 1 (5%)  Total  3 (3-3)  2022 outbreak  2 (2-2) |
| Cluster investigation | No. Syntheses that include study design  Median No. studies included per design (range) | 1  Total  11 (11-11) | 2  Total  1 (1-1)  2022 outbreak  1 (1-1) | 0 | 0 | 0 | 2  Total  2.5 (1-4)  2022 outbreak  0 (0-0) | 5 (24%)  Total  2.5 (1-11)  2022 outbreak  0.5 (0-1) |
| *In vitro* study | No. Syntheses that include study design  Median No. studies included per design (range) | 0 | 0 | 1  Total  1 (1-1)  2022 outbreak  1 (1-1) | 0 | 0 | 0 | 1 (5%)  Total  1 (1-1)  2022 outbreak  1 (1-1) |
| Press reports and new articles | No. Syntheses that include study design  Median No. studies included per design (range) | 1  Total  3 (3-3) | 1  Total  1 (1-1)  2022 outbreak  1 (1-1) | 0 | 0 | 0 | 0 | 2 (10%)  Total  2 (1-3)  2022 outbreak  1 (1-1) |

*Systematic scoping review (n=1), rapid SR (n=1), rapid systematic review meta-analysis (n=1), quantitative evidence synthesis (n=1) and mini-review (n=1)

** Total out of 21 syntheses as one historical and four new syntheses (one scoping review, one rapid review and two “other” syntheses) did not report the study designs of included syntheses.
