# Supplementary material for: Methodological and reporting quality of systematic and rapid reviews on human mpox and their utility during a public health emergency
Source: Cochrane Evid Synth Methods. 2024 Nov 15;2(11):e70005. doi: 10.1002/cesm.70005 (PMC11795912; doi:10.1002/cesm.70005)
Supplement: Supplementary file 8 — Supporting information. [file CESM-2-e70005-s001.docx]

**Supplementary 8: Number of studies that reported items in the PRISMA-Abstracts checklist**

| **Section and Topic** | **Item #** | **Checklist item** | **Prior to May 2022 (n=2)** | | | **May – Dec 2022**  **(n=24)** | | |
| --- | --- | --- | --- | --- | --- | --- | --- | --- |
|  |  |  | **No. studies that reported item** | **Total no. of applicable studies for item** | **% of studies that adhered** | **No. studies that reported item** | **Total no. of applicable studies for item** | **% of studies that adhered** |
| **TITLE** | | |  |  |  |  |  |  |
| Title | 1 | Identify the report as the respective type of evidence synthesis (E.g., systematic reviews, rapid reviews, scoping reviews). | 2 | 2 | 100 | 18 | 21 | 86 |
| **BACKGROUND** | | |  |  |  |  |  |  |
| Objectives | 2 | Provide an explicit statement of the main objective(s) or question(s) the review addresses. | 2 | 2 | 100 | 18 | 21 | 86 |
| **METHODS** |  |  |  |  |  |  |  |  |
| Eligibility criteria | 3 | Specify the inclusion and exclusion criteria for the review. | 0 | 2 | 0 | 1 | 21 | 5 |
| Information sources | 4 | Specify the information sources (e.g. databases, registers) used to identify studies and the date when each was last searched. | 1 | 2 |  | 8 | 21 | 38 |
| Risk of bias/  Critical Appraisal ** | 5 | Specify the methods to assess risk of bias in the included studies. **Specify the methods for conducting a critical appraisal of the included sources of evidence. | 0 | 2 | 0 | 2 | 21 | 12 |
| Synthesis of results | 6 | Specify the methods used to present and synthesize results. | 0 | 2 | 0 | 5 | 21 | 24 |
| **RESULTS** |  |  |  |  |  |  |  |  |
| Included studies | 7 | Give the total number of included studies and participants and summarize relevant characteristics of studies. | 1 | 2 | 50 | 12 | 21 | 57 |
| Synthesis of results | 8 | Present results for main outcomes, preferably indicating the number of included studies and participants for each. If meta-analysis was done, report the summary estimate and confidence/credible interval. If comparing groups, indicate the direction of the effect (i.e. which group is favoured).  **Summarize and present results as they relate to the review questions and objectives. | 2 | 2 | 100 | 19 | 21 | 90 |
| **DISCUSSION** | | |  |  |  |  |  |  |
| Limitations | 9 | Provide a brief summary of the limitations of the evidence included in the review (e.g. study risk of bias, inconsistency and imprecision).  **Provide a brief summary of the limitations of the scoping review process. | 0 | 2 | 0 | 2 | 21 | 10 |
| Interpretation | 10 | Provide a general interpretation of the results and important implications. | 1 | 2 | 50 | 12 | 21 | 57 |
| **OTHER** | | |  |  |  |  |  |  |
| Funding | 11 | Specify the primary source of funding for the review. | 0 | 2 | 0 | 2 | 21 | 10 |
| Registration | 12 | Provide the register name and registration number. | 1 | 2 | 50 | 1 | 21 | 5 |

*From:*  Page MJ, McKenzie JE, Bossuyt PM, Boutron I, Hoffmann TC, Mulrow CD, et al. The PRISMA 2020 statement: an updated guideline for reporting systematic reviews. BMJ 2021;372:n71. doi: 10.1136/bmj.n71

**Note: Critical appraisal refers to the process of systematically examining research evidence to assess its validity, results, and relevance before using it to inform a decision. This term is used for items 12 and 19 instead of "risk of bias" (which is more applicable to systematic reviews of interventions) to include and acknowledge the various sources of evidence that may be used in a scoping review (e.g., quantitative and/or qualitative research, expert opinion, and policy document)
